# Integrated 3D static modeling approach for evaluating the Cenomanian Bahariya reservoir in Falak Field Shushan Basin northwestern desert Egypt

**DOI:** 10.1038/s41598-026-61159-5

**Published:** 2026-07-14

**Authors:** Hady Nasser, Bassem S. Nabawy, Karrar El-Faragawy, Ahmed M. Meneisy

**Affiliations:** 1 Department of Geology, Faculty of Science, Aswan University, Aswan, Egypt; 2 Geophysical Sciences Department, National Research Centre, Dokki, Cairo, Egypt

**Keywords:** Static modeling, Albian-Cenomanian clastics, Seismic, Petrophysical, Bahariya reservoir, Shushan Basin, Environmental sciences, Solid Earth sciences

## Abstract

This study integrates petrophysical analysis, seismic interpretation, and 3D reservoir modeling to evaluate the hydrocarbon potential of the Albian-Cenomanian Bahariya Fm in the Falak Field. Seismic interpretation reveals a structurally compartmentalized field dominated by a NW-SE trending normal fault system, compartmentalizing the field into distinct up-thrown and down-thrown blocks. Time-to-depth conversion, validated by synthetic seismogram, check shot data that is used to build a velocity model, facilitated the construction of structural contour maps, identifying two primary three-way dip closure prospects. The Bahariya Fm exhibits significant thickness variation (568 ft to 861 ft), with net pay zones ranging from 47 ft to 197.5 ft. The average effective porosity is between 16.3% and 22.7%, while the average oil saturation is between 44.2% and 51.4%. Petrophysical evaluation based on well logs, cross-plots, well-to-well correlation and facies modeling (55.48% sandstone) indicates that sandstone represents the dominant modelled facies, followed by siltstone, shale, and minor carbonate/limestone facies. The sandstone-rich intervals are mainly associated with the lower Bahariya units and represent the most favorable reservoir zones. A 3D geocellular model, incorporating interpreted horizons, faults, facies distributions, and petrophysical properties (populated via Sequential Gaussian Simulation), visualizes the reservoir architecture, confirms superior reservoir quality (higher Ø_eff_, lower Sw/Vsh) within the central, northwestern, and southeastern areas, particularly in the lower fluvial sandstone units. These results prioritize the northern and southeastern structural closures for future development drilling targeting the Bahariya Fm due to optimal reservoir properties and hydrocarbon pore volumes. Model uncertainty is mainly related to sparse and uneven well control, facies distribution away from wells, log upscaling, and variogram-based property modelling. Volumetric calculations estimate the total OIIP/STOIIP of the Bahariya reservoir at approximately 160 MMSTB, with Bahariya-I contributing about 46 MMSTB and Bahariya-III contributing about 114 MMSTB. The workflow is extendable to analogue subsurface clastic reservoirs in the Northwestern Desert and North Africa.

## Introduction

 The Northwestern Desert of Egypt is one of the most prolific hydrocarbon provinces in the Western Desert, where several sedimentary basins and depressions, including Alamein, Abu Gharadig, Shushan, Ma’mura, Dabaa, Natrun, Qarun, Abu Sennan, and Gindi, host significant hydrocarbon accumulations^1–3^. Among these basins, the Shushan Basin is of particular importance because it contains several productive Cretaceous reservoirs, including the Bahariya Formation.

**Fig. 1:**
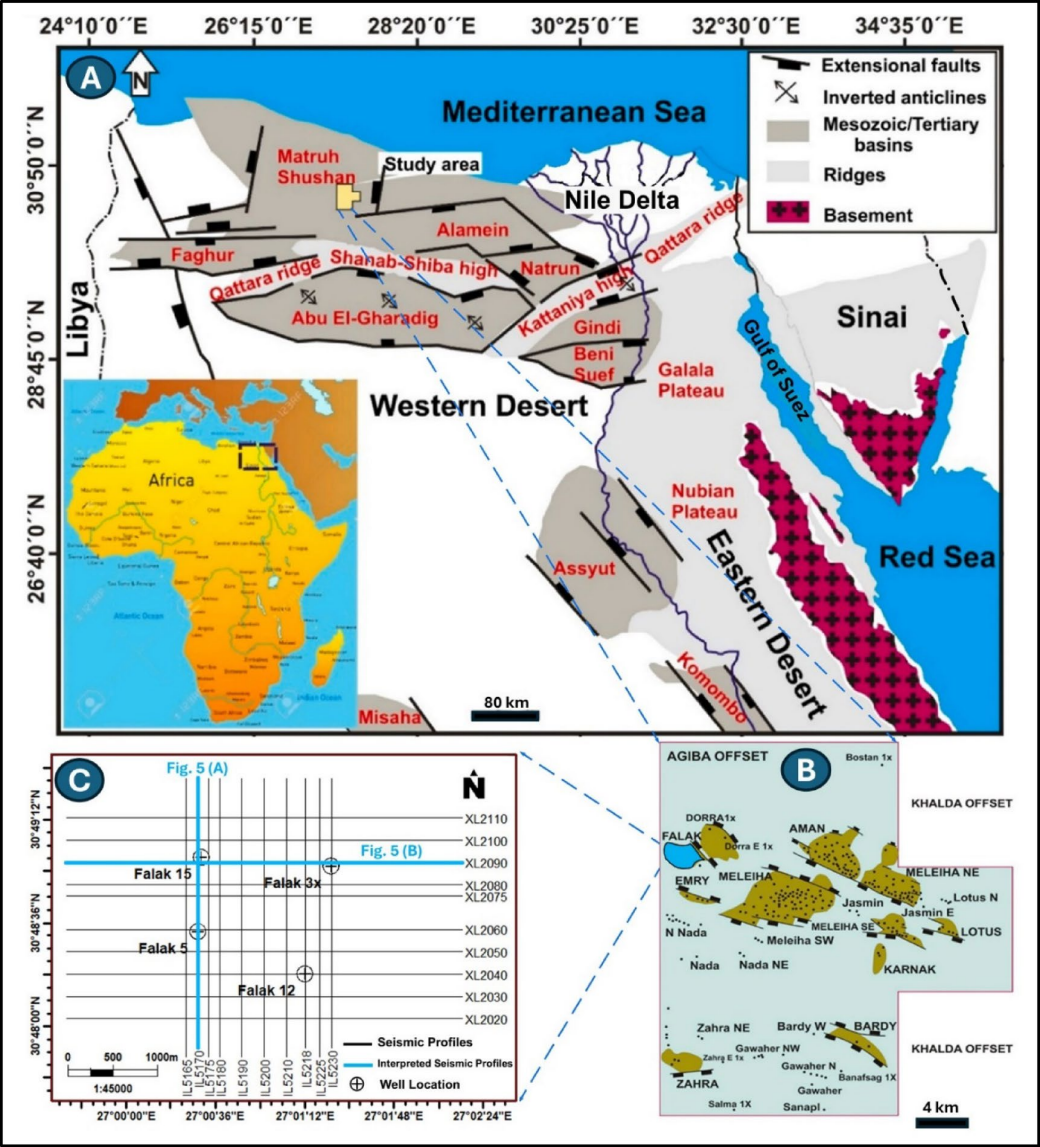
Location map of the study area in Shushan Basin, Northwestern Desert, Egypt (modified after^4,8,9^ (**A**) Base map illustrating Northern part of Egypt (**B**) Meleiha Concession including Falak Field, and (**C**) Seismic grid with studied wells.

This study focuses on the Falak Field within Agiba’s Meleiha concession in the northern Western Desert of Egypt. The concession covers approximately 700 km² and extends between latitudes 30°36′00″–30°54′00″N and longitudes 27°00′00″–27°18′00″E, nearly 65 km south of Matruh City (Fig. 1). The Falak Field covers approximately 40 km² in the northwestern part of the Meleiha Development Lease, near the central part of the Shushan Basin. It is surrounded by the Dorra Field to the northeast, the Emry Field to the south, and the Nada Field to the southeast. The field is extensively covered by subsurface data, including numerous 2D seismic profiles and several 3D seismic surveys, with available 3D seismic coverage of approximately 743 km². The presence of hydrocarbon discoveries across the Meleiha concession highlights its petroleum significance^4,5^. However, despite the regional importance of the Bahariya Formation as a major Cretaceous reservoir in the Shushan Basin, detailed field-scale integration of seismic interpretation, well-log petrophysics, lithofacies classification, and 3D static reservoir modelling remains limited for the Falak Field. Therefore, a refined seismic-to-static-modelling workflow is required to improve reservoir characterization and support future exploration and development strategies^6,7^.

The Bahariya Formation represents one of the principal hydrocarbon-bearing intervals in the Western Desert, with reservoir intervals mainly hosted within porous sandstone units^7,10^. Hydrocarbon accumulations in the Bahariya Formation are commonly associated with structurally elevated fault blocks, where reservoir sandstones occupy favorable trapping positions. Approximately 90% of hydrocarbon fields in the Western Desert have been reported to produce from the Cenomanian Bahariya sandstones^10^. Therefore, detailed characterization of the petrographic, lithofacies, and petrophysical attributes of these reservoirs is essential for reliable reservoir evaluation.

Seismic interpretation and 3D static reservoir modelling are fundamental tools for hydrocarbon exploration and field development. Their integration enables the construction of a three-dimensional subsurface framework that defines reservoir geometry, structural configuration, fault architecture, lithofacies distribution, and petrophysical-property variation. Such models support the identification of reservoir limits, optimize well placement, reduce geological uncertainty, and improve field-development planning^1^.

Previous studies have demonstrated the value of integrating seismic interpretation, well-log analysis, and 3D static modelling for evaluating the Bahariya Formation and comparable Cretaceous reservoirs in the Western Desert. Abdel-Fattah et al. (2018) developed a static reservoir model for the Bahariya reservoirs in the South Umbarka area to support oilfield development and reduce subsurface uncertainty^11^. Abdelwahhab and Raef (2020) integrated static reservoir modelling with basin modelling for the Cenomanian Bahariya reservoir in the Falak Field, using seismic and well-log data to construct structural, facies, and petrophysical models and to evaluate petroleum prospects^12^. This model can be applied to predict further petrophysical data for future development plans. Furthermore, Hassan et al. (2023) applied a 3D static modelling workflow to the Bahariya Formation in Heba Field by integrating 2D seismic interpretation and well-log analysis to model reservoir properties and reduce uncertainty in stochastic property distribution^13^. Therefore, the study is considered a more detailed study and further improvement for the previous research based on a 3D static reservoir model for the various zones of the Bahariya Formation in the Falak area, with emphasis on core-calibrated petrophysical evaluation, lithofacies classification, facies distribution, porosity–permeability modelling, water-saturation distribution, and volumetric assessment within the Bah I–Bah VI reservoir units.

A comprehensive understanding of lithofacies, mineralogical composition, petrophysical properties, and diagenetic alterations provides critical insights into reservoir flow-unit delineation and rock classification. Accordingly, this study evaluates the petrophysical properties of the Lower Cenomanian Bahariya Formation in the Shushan Basin through the construction of three-dimensional facies and petrophysical models using Petrel™ software. The models were built within a predefined three-dimensional structural and stratigraphic framework derived from the integrated interpretation of twenty 2D seismic profiles and well-log data from four wells: Falak-3X, Falak-5, Falak-12, and Falak-15. The interpreted lithological assemblage of the Bahariya Formation consists mainly of sandstones cemented by shaly and calcareous materials, interbedded with siltstone and minor limestone layers, reflecting a heterogeneous clastic depositional system. These data were incorporated into a systematic modelling workflow. A lithofacies classification is proposed for the Bahariya Formation, consisting of six distinct lithofacies identified across three wells. These include sand–silt–shale–carbonate lithofacies in the Bah I, Bah V, and Bah VI units; shale–carbonate lithofacies in the Bah II unit; and sand–silt–shale lithofacies in the Bah III and Bah IV units.

The objective is to elucidate the petrophysical properties and facies characters of the Bahariya Formation and to delineate its spatial distribution within the Falak Field. The dataset includes four digital well-log suites comprising gamma-ray, neutron, density, and resistivity logs. In addition, 2D seismic profiles, supported by check-shot data, were used to investigate the structural complexity and lateral heterogeneity of the top Bahariya surface. This surface occurs at measured depths ranging from approximately 5,900 to 6,900 ft below ground level and exhibits an apparent thickness of about 550–900 ft. The analytical workflow integrates seismic interpretation with detailed well-log analysis to improve subsurface characterization. Core data from the Falak-12 well were used to calibrate log-derived parameters, particularly through comparison between helium core porosity and neutron-density porosity measurements.

## Geologic setting

The Western Desert of Egypt forms part of the northern African passive margin and records the combined effects of Tethyan rifting, Hercynian tectonism, and later Alpine deformation. Regionally, it is subdivided into the stable shelf to the south, the unstable shelf in the central part, and the Mediterranean coastal basins to the north^14,15^. The Northwestern Desert, located within the unstable shelf, contains a complex network of Jurassic–Cretaceous rift basins, including the Shushan, Abu Gharadig, and Matruh basins. These basins developed along pre-existing basement weakness zones during the Middle Jurassic–Lower Cretaceous Tethyan rifting phase associated with the divergence between Africa and Europe^4,16^. The tectonic evolution of the area was further influenced by multiple deformation phases, including the Late Paleozoic Hercynian Orogeny, which produced structural highs such as the Sharib–Sheiba Ridge, and the Late Cretaceous–Eocene Syrian Arc folding, which generated compressional structures^17^.

The structural architecture of the Northwestern Desert is characterized by E–W to NE–SW trending rift basins that are locally offset by NW–SE wrench faults. These basins, including the Shushan Basin, formed mainly as asymmetric half-grabens during Late Jurassic–Early Cretaceous extension related to Neo-Tethys rifting^18,19^. The Shushan Basin is considered one of the largest coastal basins in the northern Western Desert and is bounded by faults that were reactivated during Syrian Arc inversion, producing tilted fault blocks and faulted anticlines^4,20^. Its structural evolution can be summarized in three main phases: Jurassic rifting, Cretaceous passive-margin subsidence, and Late Cretaceous–Tertiary compression^21^.

The Meleiha concession, within the Agiba Development Lease, lies in the central part of the Shushan Basin. The Falak Field, discovered in 1983, is located in the northwestern part of the Meleiha Development Lease and is structurally expressed as a NW–SE trending anticline with four-way closure, segmented by normal and strike-slip faults^4^. The field is surrounded by the Dorra Field to the northeast, the Emry Field to the south, and the Nada Field to the southeast, reflecting its location within a productive hydrocarbon province of the Meleiha concession.

**Fig. 2:**
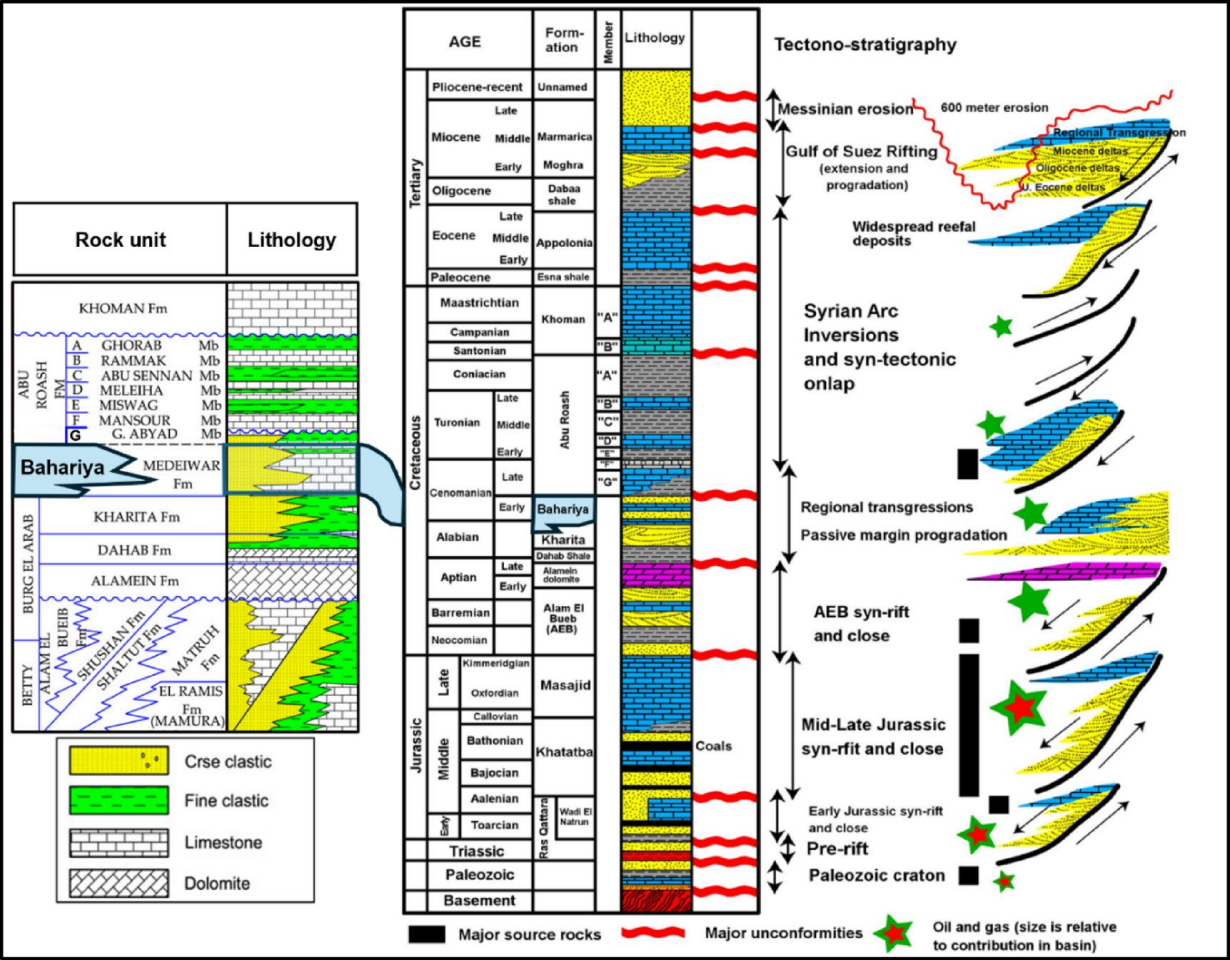
Lithostratigraphic section of Shushan Basin representing the tectonic and stratigraphic development of its sedimentary sequence^25,26^.

The basin fill includes Jurassic syn-rift clastics of the Khatatba Formation, Cretaceous post-rift marls of the Alam El Bueib Formation, and Cenomanian–Turonian carbonates of the Abu Roash Formation (Fig. 2)^4^. The Cenomanian Bahariya Formation is a fluvio-marine succession and represents a key hydrocarbon reservoir in the Northwestern Desert. Stratigraphically, it unconformably overlies the Albian Kharita Formation and underlies the Abu Roash Formation, with a gradational upper contact marked by a carbonate-rich interval^22^. The formation comprises two main members: the Lower Bahariya, also known as the Razzak Member, which consists mainly of coarse-grained fluvial sandstone, and the Upper Bahariya, which consists of mixed carbonate, siltstone, and shale deposited in estuarine to shallow-marine settings^23,24^. Reservoir quality within the Bahariya Formation is strongly affected by diagenetic processes; quartz overgrowths commonly reduce porosity, whereas carbonate cementation may enhance reservoir compartmentalization^3^.

## Materials and methods

**Fig. 3:**
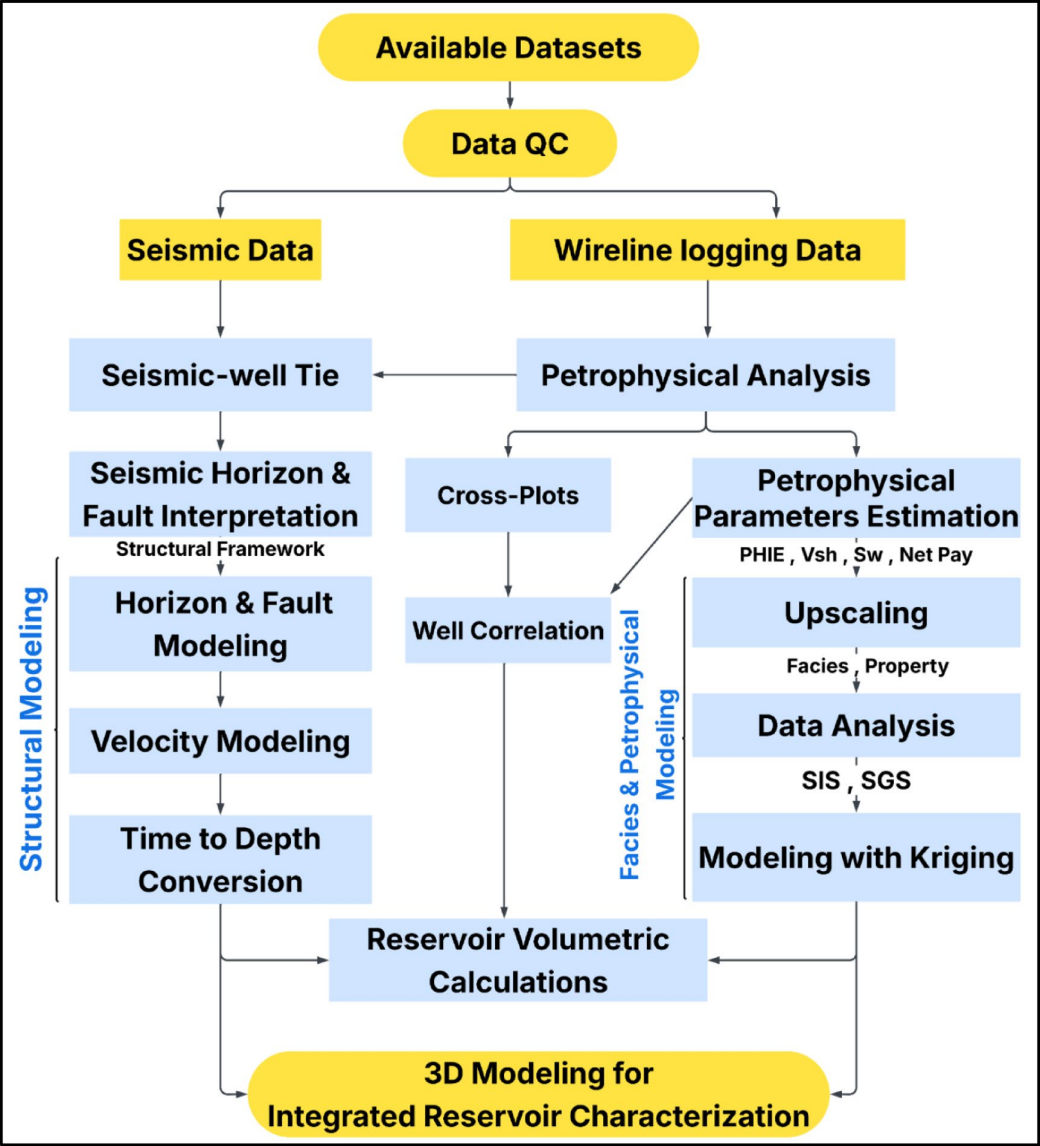
Workflow presenting the applied flowchart for methods and techniques that have been employed for modeling the Bahariya reservoirs.

The objectives have been achieved through a structure of workflow (Fig. 3) integrating the available geophysical and geological datasets. The analysis comprised twenty 2D seismic profiles, formation-top picks, and a full set of well log data (caliper, gamma-ray, micro-spherically focused, deep and shallow resistivity, neutron, density, and photoelectric factor logs) from four wells penetrating the Bahariya Fm (Falak-3X, Falak-5, Falak-12, and Falak-15). All data were provided by Agiba Oil Company with authorization from the Egyptian General Petroleum Corporation (EGPC).

###  Seismic data

#### Seismic data quality check and workflow

The interpretation workflow initiated by delineating the formation tops of stratigraphic horizons and mapping fault geometries to characterize the dominant structural framework of the Falak Field. Two specific horizons, the AR/G Mb and the Bahariya Fm, were identified and picked across seismic data. Depth-structure contour maps were subsequently generated for these horizons, integrating interpreted fault trends to visualize subsurface deformation patterns. Horizon identification was defined by tying seismic reflections to well tops derived from wireline logs of the four studied wells, ensuring confirm time-to-depth calibration. Seismic interpretation and mapping were performed using Petrel™ 2018. This workflow has been widely published by numerous authors^2,27–31^.

Interpreting the seismic data for both stratigraphic horizons and structural elements constitute a fundamental step in supporting the construction of the 3D subsurface geological model and in detection of promising reservoirs. Seismic data, perceived as strong reflectors, is analyzed to map faults, channel systems, reservoir bodies, and associated depositional or structural features. Calibration of seismic events with subsurface formation was achieved through well-to-seismic, utilizing 1D synthetic seismic modelling to ensure accurate correlation for the various wells. This integration enhances confidence and correlates the interpreted seismic profiles with the subsurface formation tops^32^.

Faults and stratigraphic horizons are interpreted manually and subsequently validated using twenty 2D seismic profiles (Fig. 1C). The interpretation methodology integrated seismic data with well-logs to establish coherent stratigraphic sequences and correlate seismic reflections with subsurface formation tops, derived from the four wells and superimposed on seismic sections to evaluate the attributes of the formations. The interpreted seismic profiles are then converted into depth contour and structural maps in time domain, enabling detailed assessment of the structural configuration and its implications for reservoir distribution.

#### Seismic-well tie

It is utilized to establish a correlation between the depths of the formation tops, as indicated by the well log data, and the horizons identified in the 2D seismic profiles. The estimation is derived from the borehole data and associated with a specific seismic zone. The generated seismogram serves as primary connection between borehole data and seismic, functioning as the principal instrument for geological interpretation. This will be corroborated by check-shot data as a substitute for the synthetic seismogram. When employing combined density and acoustic logs for the analyzed borehole, the reflection coefficient is convolved with the wavelet to generate the synthetic seismic trace.

**Fig. 4:**
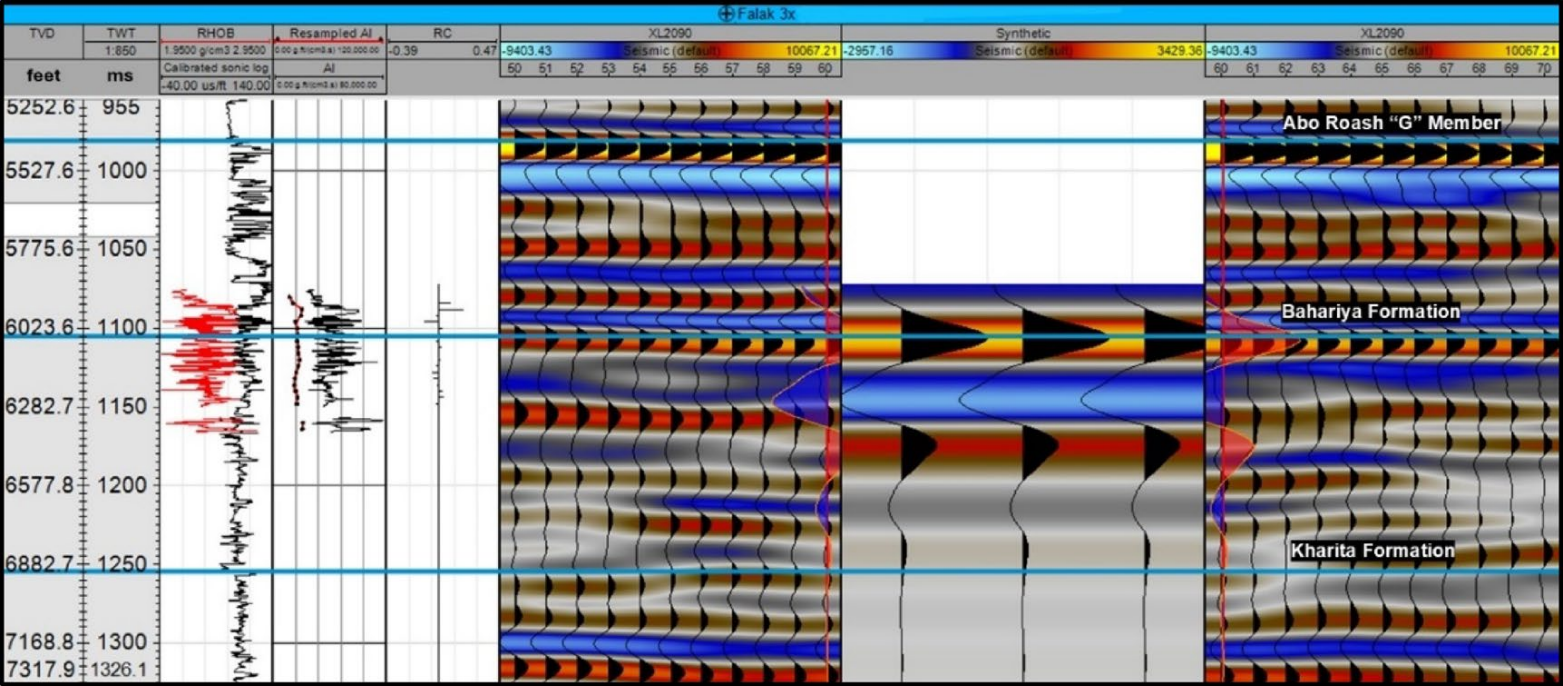
Synthetic seismogram of the Falak-3x well displaying the seismic-well tie by check-shot data.

Synthetic seismogram has been prepared for the well that crossed the seismic profiles. Generally, tying between the synthetic seismogram and the available seismic data was applied at a high certainty level (Fig. 4). As an example, the synthetic seismogram of the Falak-3x well indicates an acceptable Correlating with the seismic section profile (XL2090) at the studied time domain, to select the reflectors of interest along the Field. Results show that reflectors representing Bahariya Fm lies around − 1100 ms while shallower Abo Roash “G” Mb lies around 975 ms.

The interpretation of seismic data from subsurface sequences reveals geological and structural features, represented on seismic profiles interpreted as continuous horizons. Conversely, faults are characterized as discontinuities within these horizons.

**Fig. 5:**
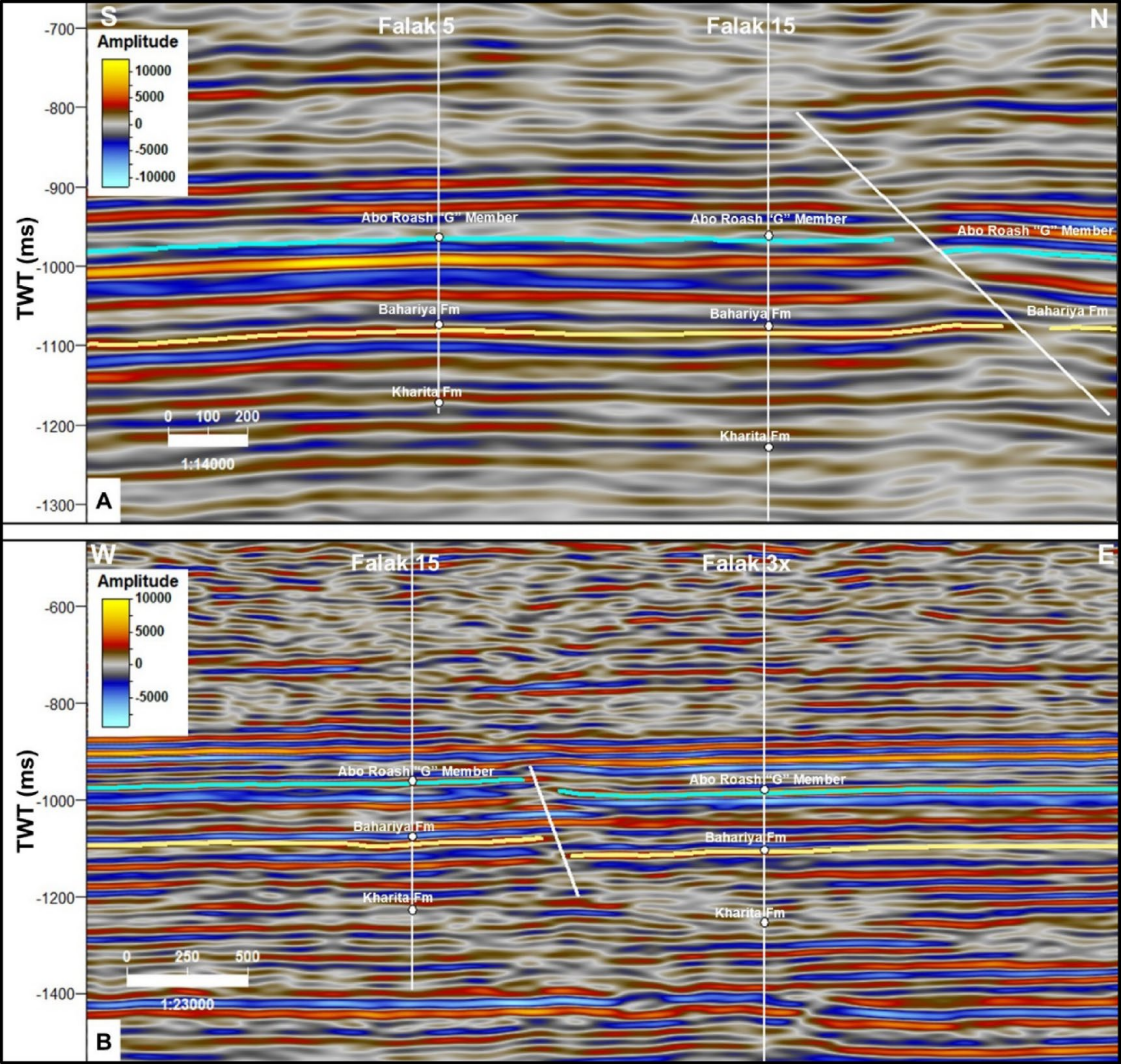
(**A**) Interpreted S-N trending 2D seismic profile IL5170 and (**B**) Interpreted W-E trending 2D seismic profile XL2090 (the location of seismic lines are presented in Fig. 1C).

**Fig. 6:**
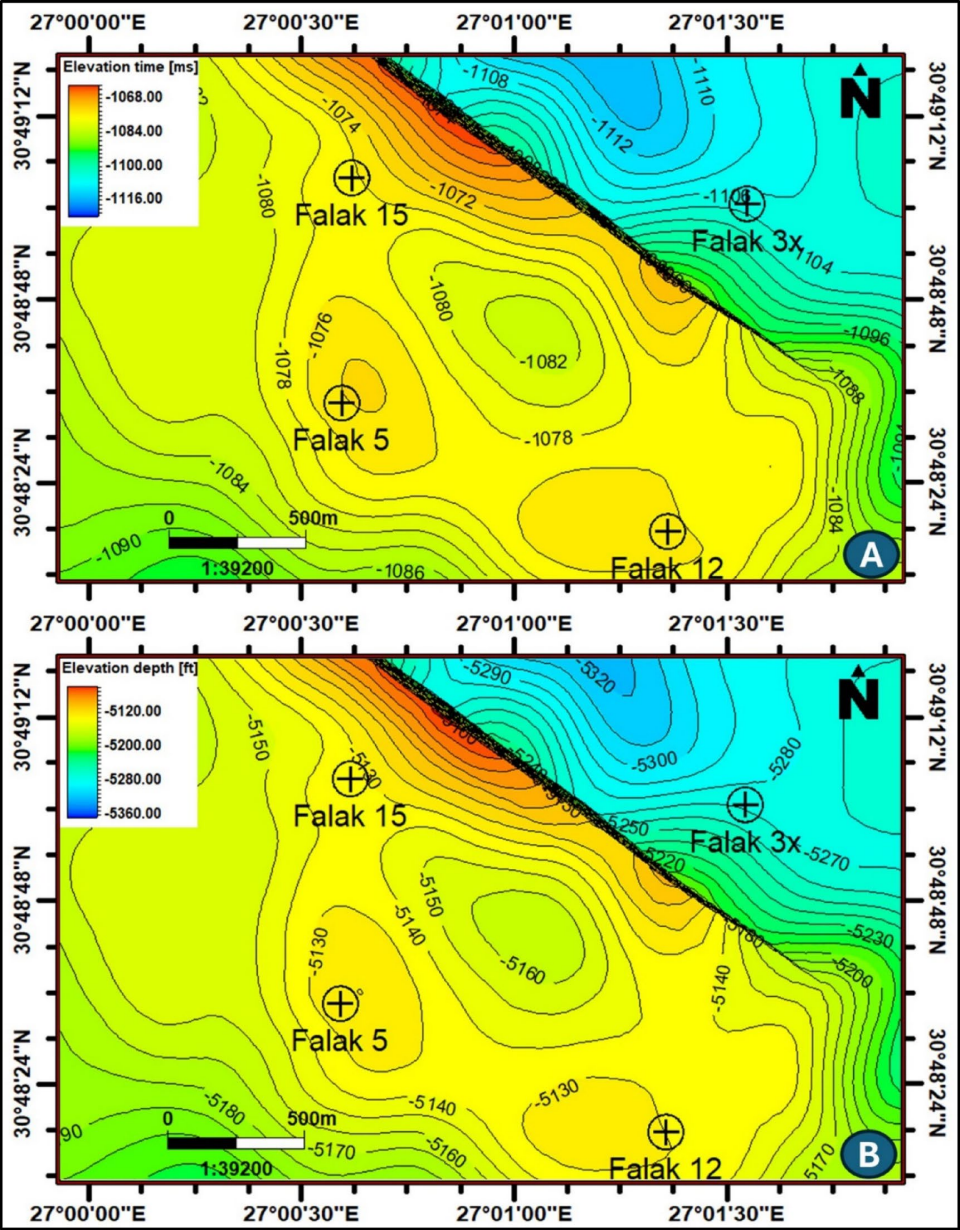
(**A**) The structure contour map in the time domain and (**B**) I the depth domain to the top surface of the Bahariya Fm.

The seismic analysis of the Falak structure indicates that the E-W trending three-way dip closures are affected by faults. The fault exhibits a predominantly moderate to steep dip (Fig. 6). The IL-5170 and XL-2090 seismic sections exhibit a primary normal fault. In other seismic sections, we observe the presence of normal faults exclusively, indicating a straightforward structural pattern, as illustrated in Fig. 5. Normal faulting is the dominant structural style in the Falak Field and across the Meleiha concession. Several prospects are represented in two-way time and depth maps as purely structural prospects, presenting new challenges for field development, as delineated by Azzam^5^.

The normal fault is found to be as a step-fault zone structure dipping from 60° to 70° with NW-SE direction trend. This structure suggests that the field has been controlled structurally by compressive inversion events from the compression and shear of the Upper Cretaceous, Mid-Cretaceous uplifting, and the Lower Cretaceous rift. It is stated that the Middle Cretaceous Bahariya and Kharita Formations and the Upper Cretaceous Abu Roash were influenced by normal faulting. Then constructing Two-Way Time (TWT) and True-Vertical Depth (TVD) structural contour maps for the top Bahariya Fm (Fig. 6). These time and depth structural maps show that Falak Field is influenced by the NW-SE trending Fault located to the northern portion of the field.

The time structural map of the top surface of the Bahariya Fm decreases in the center and northwestern portions of the area varying from − 1062 ms to – 1086 ms (Fig. 6a). From this figure, it is shown that the maximum TWT values vary from − 1092 ms to – 1122 ms in the northeastern part. These high and low time anomalies are recorded in structural highs and lows occur within the downthrown and upthrown blocks, which are segmented by the main NW–SE-trending normal fault. The time structure maps of the Bahariya Fm, in conjunction with the velocity model, are utilized to convert reflection times to depths, with the objective of constructing structure depth maps (Fig. 6b).

The depth structure map of the upper surface of the Bahariya Fm, derived from 2D seismic data, indicates that it is structurally elevated (Fig. 6b). The maximum depth values range from − 5250 ft to − 5370 ft in the northeastern region, whereas the minimum depth values are found in the central and northwestern areas, ranging from − 5050 ft to − 5190 ft.

Two primary prospects exist that could advance the field by drilling additional exploratory wells. The initial feature identified on the Bahariya map is a significant three-way dip closure located on the upthrown side of the main fault in the northwestern side of the field. The second prospect in Bahariya is a minor three-way dip closure situated on the downthrown side of the main fault in the northeastern section of the field.

### Wireline logging workflow

Comprehensive set of logs was utilized from four wells in the Falak Field (Falak-3x, Falak-5, Falak-12, and Falak-15) penetrating the Bahariya Fm. The available datasets included gamma-ray, density, resistivity (shallow and deep), neutron, and photoelectric factor (PEF) logs. Petrophysical interpretation involved lithological classification supported by a series of X–Y cross-plots illustrating the petrophysical parameters across the studied wells^33,34^.

For this purpose, the well-log datasets were processed and evaluated using Techlog™, a specialized Schlumberger software designed to manage petrophysical data and generate depth-dependent log displays and X–Y plots. Borehole data were incorporated during petrophysical data processing in accordance with established Schlumberger standards and correction charts^25,29,35,36^. This integrated approach ensured that the derived petrophysical parameters accurately reflected formation characteristics and were suitable for reservoir characterization and modelling workflows.

#### Petrophysical parameters estimation

##### Lithology determination

Multiple methods were employed in parallel to characterize lithology, including density-neutron cross-plots, which have been widely recommended for reliable lithological discrimination^36^.

##### Shale volume (Vsh) estimation

Accurate determination of shale volume is a critical step in well-log analysis, as shale content significantly influences the well-log measurements. Among the available tools, gamma-ray log provides one of the most effective means to detect shale intervals and estimating shale distribution within the formation^37^.

Shale volume within Bahariya Fm was quantified using several established calculation techniques^38,39^. Mostly the measured values were reported as follows.

1 $${{\mathrm{V}}_{{\mathrm{sh}}}} = 0.083 \times \left( {{2^{3.7 \times \left( {{\mathrm{G}}{{\mathrm{R}}_{{\mathrm{Log}}}} - {\mathrm{G}}{{\mathrm{R}}_{\min }}} \right)/\left( {{\mathrm{G}}{{\mathrm{R}}_{{\mathrm{max}}}} - {\mathrm{G}}{{\mathrm{R}}_{\min }}} \right)}} - 1} \right)$$

##### Porosity estimation

The total (∅_T_) and the effective porosity (∅_eff_) and values were calculated using the neutron-density values as follows^25^.

2 $${\emptyset _{\mathrm{T}}} = {\text{ }}\left( {{\emptyset _{{\mathrm{NLog}}}} + {\text{ }}{\emptyset _{{\mathrm{DLog}}}}} \right){\mathrm{/2}}$$

3 $${\emptyset _{{\mathrm{eff}}}} = {\emptyset _{\mathrm{T}}}\left( {{\text{1 }} - {\text{ Vsh}}} \right)$$

where Ø_DLog_ and Ø_NLog_ are the density and neutron porosities, respectively.

##### Water saturation (S_w_) estimation

Owing to the heterogeneity and complicated nature of the reservoir lithologies and petrophysical characteristics, several empirical equations are commonly used to estimate water saturation (S_w_). For argillaceous clastic reservoirs characterized by relatively high shale content (Vsh > 10%), such as the Bahariya Fm, the Simandoux and Indonesian equations are considered particularly suitable due to their ability to account for shale conductivity effects.

4 $${\text{Sw }} = {\text{ }}\left( {{\mathrm{a}}.{\text{ Rw}}} \right)/\left( {{\mathrm{2}}.{\text{ }} \emptyset {\mathrm{m}}} \right).{\text{ }}\{ {\text{SQRT }}[\left( {{\mathrm{Vsh/Rsh}}} \right){\text{2 }} + {\text{ }}(({\mathrm{4}} * \emptyset {\mathrm{m}})/\left( {{\mathrm{a}}.{\mathrm{Rw}}.{\text{ RT}}} \right))]{\text{ }} - {\text{ }}({\mathrm{Vsh/Rsh}})\}$$

##### Oil saturation (S_O_) estimation

Oil saturation (S_O_) represents the portion of the pore volume that is already occupied by oil and is typically derived as a complementary value for water saturation^40–42^. The principal equation utilized for the computation of oil saturation is given by:

5 $${{\mathrm{S}}_{\mathrm{O}}} = {\text{ 1 }} - {\text{ }}{{\mathrm{S}}_{\mathrm{w}}}$$

where S_O_ and S_w_ denote the oil and water saturations of the uninvaded zone, respectively.

The determined petrophysical parameters for the Falak Field (shale volume (Vsh, %), effective porosity (∅_eff_, %), net-pay thickness, as well as the oil and water saturations (S_O_ and S_W_, %), respectively) are summarized in Table 1. Petrophysical processing and net-pay identification were conducted by applying cutoff values of 50% for Vsh, 65% for Sw, and 12% for the effective porosity according to reports that were provided by Agiba Oil Company with authorization from the Egyptian General Petroleum Corporation (EGPC).

Based on its identified hydrocarbon potential from integrated seismic and well-log interpretation, the Albian-Cenomanian Bahariya Fm was selected for detailed petrophysical and reservoir characterization. The cumulative gross thickness of the Bahariya Fm, encompassing its units, ranges from 568 ft in the Falak-5 well located in the southwestern sector of the field to 861 ft in the Falak-3x well situated in the northeastern side of the field. The petrophysical characterization of the studied field stated that net-pay thickness, the net to gross (N/G), the water saturation (S_w_), shale volume (V_sh_), and the effective porosity values (Ø_eff_), indicates that the shale volume ranging from 23.9% in Falak-12 well in the southeastern of the field up to 38% in the Falak-3x well at the northeastern parts of the field. This net-pay thickness is represented by thick, clean sandstones with high deep resistivity, high neutron porosity, and low gamma-ray. The effective porosity is more than 16.3% in Falak-5 well up to 22.7% in Falak-12 well, while the oil saturation is more than 44.2% up to 51.4%. To estimate the net-pay thickness, cutoff values were utilized based on the well testing data. A total net-pay thickness is estimated to range from 47 to 197.5 ft, as indicated in Table 1.

Properties of these promising reservoirs with their relatively high oil saturation resulted in excellent BPV values (Ø_eff_*H) (the Bulk pore volume) varying between 7.66 and 37.13, The Bulk water volume BWV values (Ø_eff_ *Sw) vary between 0.091 and 0.114 and The Hydrocarbon pore volume HCPV values (Ø_eff_ *So*H) vary between 3.386 and 19.085. This estimated reservoir and petrophysical properties are listed in Table 1. Averages of the various petrophysical parameters of the differentiated zones indicate that Bahariya V and VI have the best reservoir quality and net-pay thicknesses (Table 2).

 Then the fluctuation of petrophysical characteristics and reservoir parameters of the Bahariya Fm is chiefly ascribed to its structurally intricate configuration in the Falak Field. Therefore, to ascertain the lithological composition and identify the prospective reservoir zones, a series of X–Y plots and litho-saturation cross plots are given and analyzed in the subsequent Sect^43^.

**Fig. 7:**
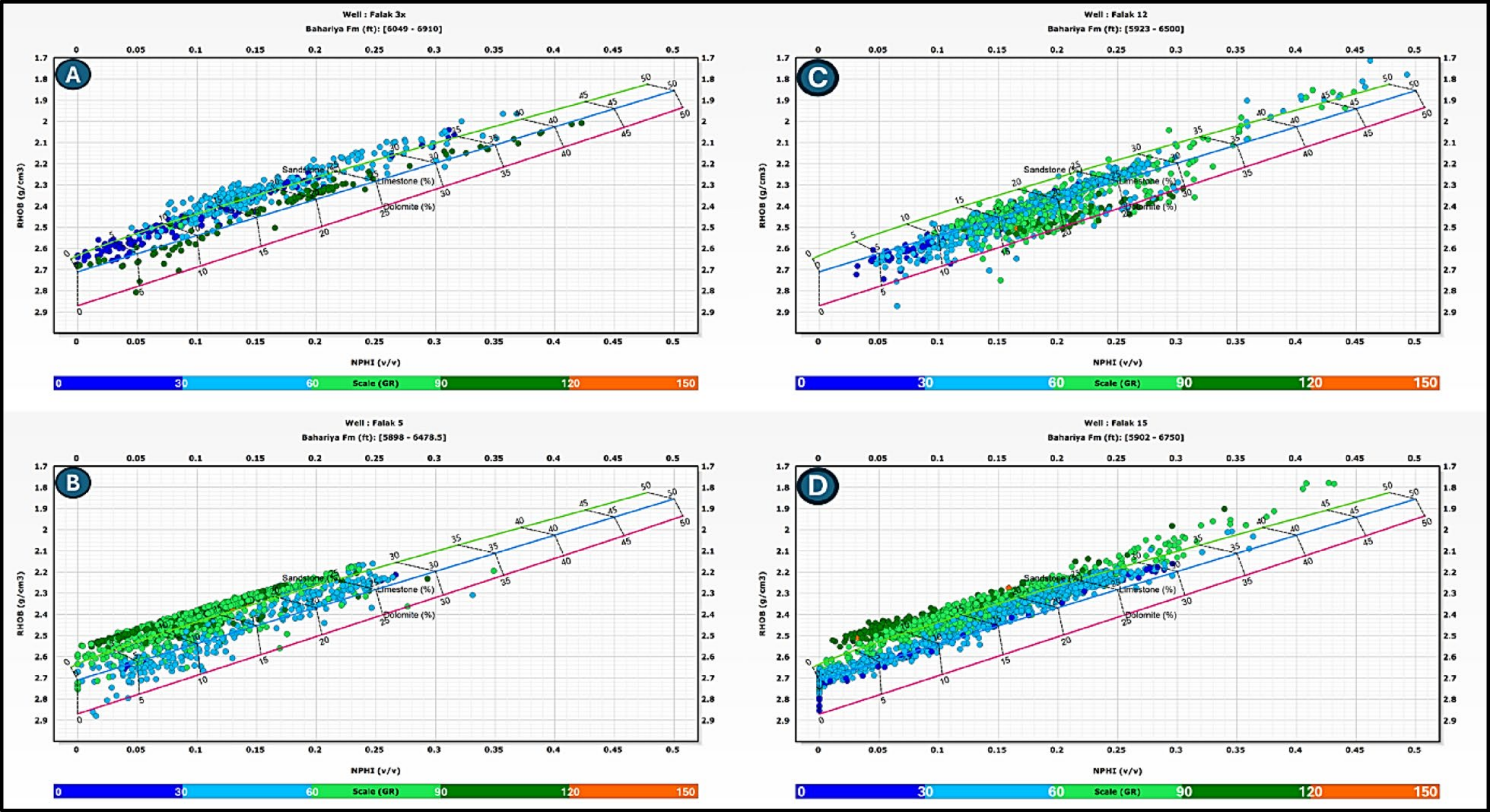
Neutron (NPHI) versus density (RHOB) cross-plots represent the lithology components of the studied Bahariya Fm in Falak-3x (**A**), Falak-5 (**B**), Falak 12 (**C**), and Falak 15 (**D**) wells.

**Fig. 8:**
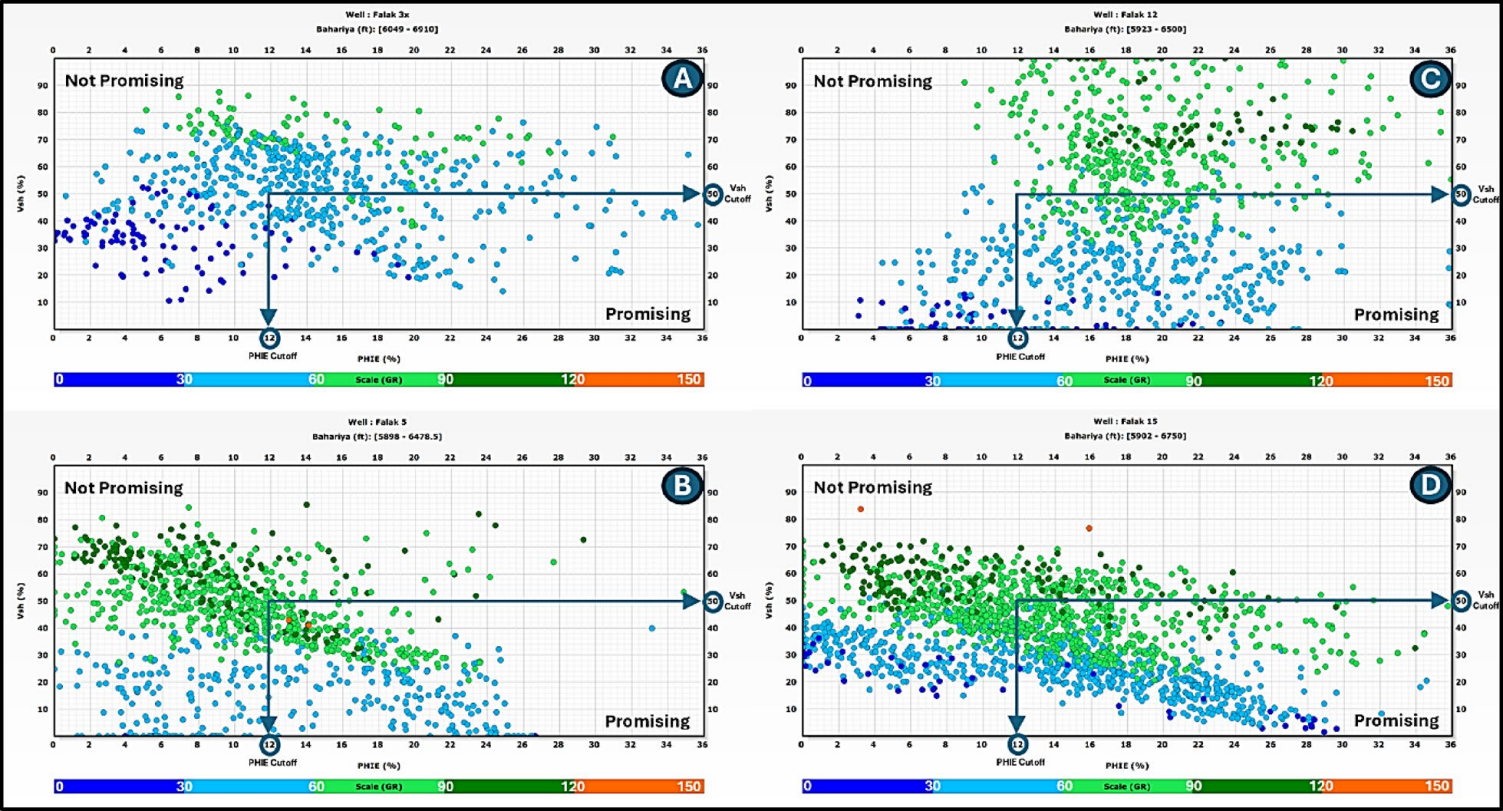
Cross-plot of the shale volume (Vsh) versus the effective porosity (PHIE) along with the gamma-ray bar to determine the cutoff values of the Bahariya Fm in Falak-3x (A) and Falak-5 (B), Falak 12 (C) and Falak 15 (D) wells.

**Fig. 9:**
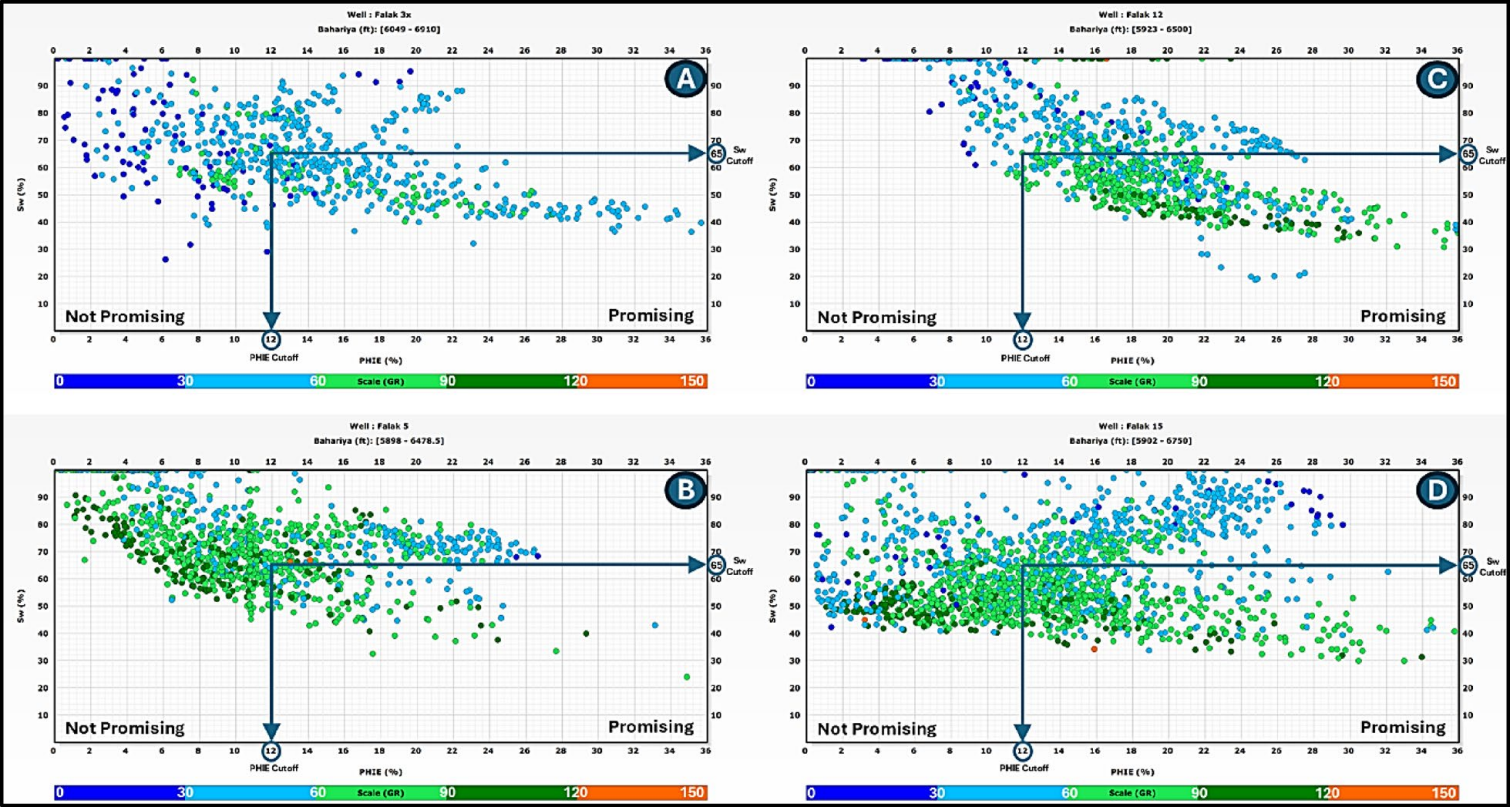
Cross-plot of the water saturation (Sw) versus the effective porosity (PHIE) along with the gamma-ray bar for determining the cutoff values of the Bahariya Fm in Falak-3x (**A**) and Falak-5 (**B**), Falak 12 (**C**) and Falak 15 (**D**) wells.

A set of X–Y plots including the density-neutron (RHOB vs. NPHI) plots were constructed along with the gamma-ray (GR) values (Fig. 7) and cutoffs determination plots (Figs. 8 and 9).

**Table 1:** The reservoir petrophysical parameters of the Bahariya Fm estimated from the well log data.

Well	Zones		Top (ft)	Bottom (ft)	Gross (ft)	Net (ft)(H)	*N*/G (frac.)	Vsh (%)	Øeff (%)	Sw (%)	So (%)	BPV= Øeff*H	BWV=Øeff(frac.)* Sw(frac.)	HCPV= Øeff*So*H
Falak 3x	Bahariya Fm (undifferentiated)		6049	6910	861	51	0.059	(38.0)	(22.4)	(48.8)	(51.2)	11.424	0.109	5.849
Falak 5	Bahariya Fm	Bahariya I	5898	5996	98	16	0.163	36.8	16.9	56	44	2.704	0.095	1.19
		Bahariya II	5996	6049	53	2.5	0.047	34.2	21.9	49.4	50.6	0.548	0.108	0.277
		Bahariya III	6049	6084	35	4	0.114	40.1	15.3	50.6	49.4	0.612	0.077	0.302
		Bahariya IV	6084	6115	31	5	0.161	27.7	17.6	54.8	45.2	0.88	0.096	0.398
		Bahariya V	6115	6230	115	8.5	0.074	31.3	15	58.2	41.8	1.275	0.087	0.533
		Bahariya VI	6230	6478.5	236.5	11	0.047	33.4	15.1	58.3	41.7	1.661	0.088	0.693
		Total (average)	5898	6478.5	568.5	47	0.083	(34.2)	(16.3)	(55.8)	(44.2)	7.661	0.091	3.386
Falak 12	Bahariya Fm	Bahariya I	5923	6020	97	17.5	0.18	35.9	18.8	39.6	60.4	3.29	0.074	1.987
		Bahariya II	6020	6064	44	9	0.205	23.7	27.4	40.4	59.6	2.466	0.111	1.47
		Bahariya III	6064	6118	54	16	0.296	23.7	23.8	44.5	55.5	3.808	0.106	2.113
		Bahariya IV	6118	6145	27	3	0.111	21.4	18.1	58.8	41.2	0.543	0.106	0.224
		Bahariya V	6145	6261	116	11	0.095	23	17.4	56.4	43.6	1.914	0.098	0.835
		Bahariya VI	6261	6500	239	59	0.247	20.7	24.1	55	45	14.219	0.133	6.399
		Total (average)	5923	6500	577	115.5	0.2	(23.9)	(22.7)	(50.4)	(49.6)	26.219	0.114	13.004
Falak 15	Bahariya Fm	Bahariya I	5902	6000	98	44	0.449	34.8	18.2	53.5	46.5	8.008	0.097	3.724
		Bahariya II	6000	6050	50	13	0.26	30.3	22.3	46.6	53.4	2.899	0.104	1.548
		Bahariya III	6050	6085	35	15.5	0.443	40.9	17.4	50.4	49.6	2.697	0.088	1.338
		Bahariya IV	6085	6110	25	8	0.32	41.6	16.8	51.4	48.6	1.344	0.086	0.653
		Bahariya V	6110	6232	122	41.5	0.34	37.6	18.4	49.5	50.5	7.636	0.091	3.856
		Bahariya VI	6232	6750	518	75.5	0.146	34.6	19.3	45.3	54.7	14.572	0.087	7.971
		Total (average)	5902	6750	848	197.5	0.233	(35.8)	(18.8)	(48.6)	(51.4)	37.13	0.091	19.09

where H is the thickness of the net-pay zone, V_sh_ is the shale volume, S_O_ and S_W_ are the hydrocarbon and water saturations, respectively, ∅_eff_ is the effective porosity, N/G is the net to gross ratio, BPV is the bulk pore volume, BWV is the bulk water volume, and HCPV is the hydrocarbon pore volume.

**Table 2:** The average petrophysical parameters of the differentiated Bahariya zones in the various wells based on the well log data.

Zones	Gross (ft)	Net (ft)(H)	*N*/G (frac.)	Vsh (%)	Øeff (%)	Sw (%)	So (%)	BPV= Øeff*H	BWV= Øeff*Sw	HCPV= Øeff*So*H
Bahariya I	98	16	0.163	36.8	16.9	56	44	2.704	0.095	1.19
	97	17.5	0.18	35.9	18.8	39.6	60.4	3.29	0.074	1.987
	98	44	0.449	34.8	18.2	53.5	46.5	8.008	0.097	3.724
Average	97.67	25.83	0.26	35.8	18.0	49.7	50.3	4.67	0.09	2.30
Bahariya II	53	2.5	0.047	34.2	21.9	49.4	50.6	0.548	0.108	0.277
	44	9	0.205	23.7	27.4	40.4	59.6	2.466	0.111	1.47
	50	13	0.26	30.3	22.3	46.6	53.4	2.899	0.104	1.548
Average	49.00	8.17	0.17	29.4	23.9	45.5	54.5	1.97	0.11	1.10
Bahariya III	35	4	0.114	40.1	15.3	50.6	49.4	0.612	0.077	0.302
	54	16	0.296	23.7	23.8	44.5	55.5	3.808	0.106	2.113
	35	15.5	0.443	40.9	17.4	50.4	49.6	2.697	0.088	1.338
Average	41.33	11.83	0.28	34.9	18.8	48.5	51.5	2.37	0.09	1.25
Bahariya IV	31	5	0.161	27.7	17.6	54.8	45.2	0.88	0.096	0.398
	27	3	0.111	21.4	18.1	58.8	41.2	0.543	0.106	0.224
	25	8	0.32	41.6	16.8	51.4	48.6	1.344	0.086	0.653
Average	27.67	5.33	0.20	30.2	17.5	55.0	45.0	0.92	0.10	0.43
Bahariya V	115	8.5	0.074	31.3	15	58.2	41.8	1.275	0.087	0.533
	116	11	0.095	23	17.4	56.4	43.6	1.914	0.098	0.835
	122	41.5	0.34	37.6	18.4	49.5	50.5	7.636	0.091	3.856
Average	117.67	20.33	0.17	30.6	16.9	54.7	45.3	3.61	0.09	1.74
Bahariya VI	236.5	11	0.047	33.4	15.1	58.3	41.7	1.66	0.088	0.693
	239	59	0.247	20.7	24.1	55	45	14.22	0.133	6.399
	518	75.5	0.146	34.6	19.3	45.3	54.7	14.57	0.087	7.971
Average	331.17	48.50	0.15	29.6	19.5	52.9	47.1	10.15	0.10	5.02

Denotations as in Table 1. Data of Falak 3X well is not included, where the Bahariya is not differentiated in this well.

#### Cross plots

##### RHOB-NPHI plot

The density-neutron plot of the Bahariya reservoir sequence over all wells demonstrates that sandstone predominates as the primary lithology of the formation, accompanied by minor calcareous and shale intercalations. The majority of the tested locations exhibit porosity exceeding the threshold value of 12%, reaching as high as 45%. Elevated gamma-ray (GR) readings within the Bahariya Fm are attributed to the high siltstone content within the rock phase and the presence of uranium enriched in the water content. These increased GR values are enhanced by the occurrence of numerous shale streaks, particularly within the upper Bahariya layers, which were deposited under estuarine to shallow-marine conditions. This depositional interpretation is consistent with previous studies that were documented by various authors^24,44–46^.

In addition, X-ray diffraction (XRD) analyses revealed a distinctive mineralogical assemblage in the Bahariya Fm of the Shushan Basin, characterized by relatively high proportions of K-feldspar (up to 19.2%), glauconite (up to 11.2%), and pyrite (up to 8.1%). The enrichment of potassium- feldspars and accessory minerals contributes to reduced resistivity values and heightened GR readings, even inside oil-bearing zones. It is advisable in the next exploration plan to utilize spectral gamma logging to facilitate the estimation of corrected GR values.

##### Vsh-PHIE plot

The shale volume cutoff differentiates sand readings from shale readings. This approach facilitates the recognition of total sand intervals by correlating effective porosity with shale content derived from the analyzed wells and their associated gamma-ray log readings at varying depths^47^. Analysis of the cross-plot indicates that a shale volume cutoff (Vshc) of 50% effectively separates reservoir-quality rocks from non-reservoir intervals. Accordingly, intervals with shale volumes exceeding 50% are classified as non-reservoir, whereas those with shale volumes below this threshold are considered potential reservoirs. Furthermore, an effective porosity cutoff was applied to differentiate between productive porous sandstones and tight, non-productive sand intervals, representing the minimum porosity required to allow efficient oil flow. The multi-well cross plot of the Vsh-PHIE and Sw-PHIE, both accompanied by the gamma-ray scale (Figs. 8 and 9), was utilized to establish the porosity cutoff. The data indicate that an effective porosity of 12% serves as the threshold for distinguishing between reservoir (promising) and non-reservoir (not promising) horizons.

##### S_W_-PHIE plot

A water saturation cutoff was applied to differentiate net pay (oil-bearing) from non-pay (water-bearing) intervals within the porous reservoir. This distinction was determined using a water saturation-effective porosity cross plot, with lithology corroborated by the gamma-ray log, as shown in Fig. 9. Intervals with water saturation above 65% are classified as water-wet or non-productive, and those with water saturation below 65% are deemed oil-wet or productive net-pay zones.

In summary, the majority of profitable hydrocarbon pay zones have a water saturation below the cutoff value of 65% and an effective porosity beyond the cutoff value of 12%, along with minimal clay content.

#### Well to well correlation

**Fig. 10:**
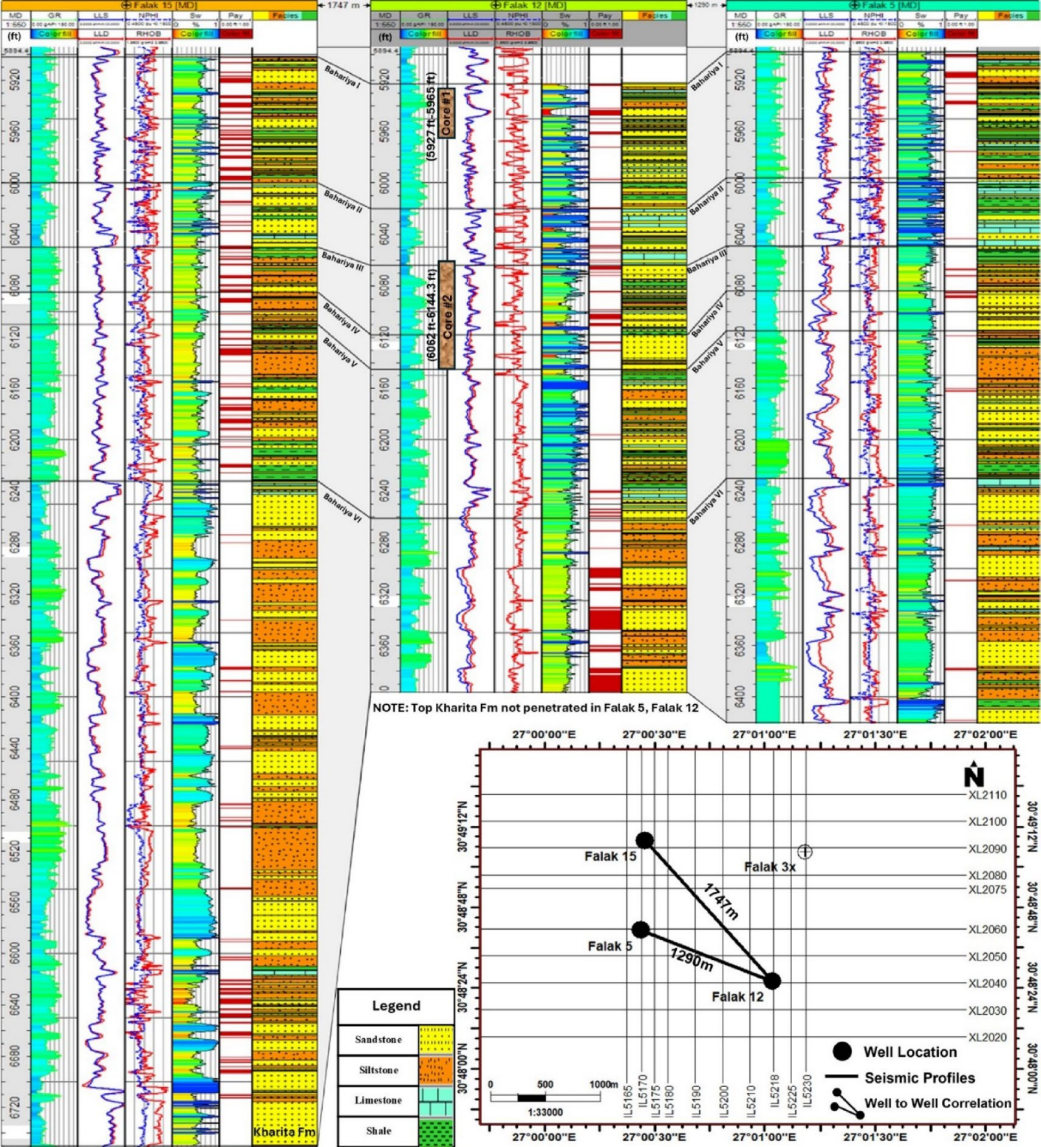
Well-to-well correlation chart between the various Bahariya units (I, II, III, IV, V, VI) in Falak 5, Falak 12, Falak 15 wells based on the well log data of the Falak wells. The lower boundary of Bahariya VI in Falak-5 and Falak-12 wells represents the total penetrated depth (TPD), where the top Kharita Formation was not penetrated.

The well-to-well correlation chart (Fig. 10) illustrates thickness variation of the Bahariya Fm and its subdivisions. The Bahariya Fm exhibits consistent thickness, which increases towards the northern and northeastern regions where the Falak-15 and Falak-3x wells are situated, due to an elevated rate of structural subsidence.

The correlation charts are designed to track variations in lithological characteristics or any disruptions in depositional continuity. It demonstrates the equivalence of stratigraphic units and displays variations in thickness. The correlation profile starts from Falak-15 well, then passes through Falak-12 with a horizontal distance of 1747 m and ends at Falak-5 well with a distance 1290 m. This correlation shows all units have no such big variation except in Bahariya VI-unit thickening towards the northwestern parts increases from a penetrated thickness of 236.5 ft in Falak-5 well and 239 ft in Falak-12 to 518 ft in Falak-15, as shown in Fig. 10; Table 1. In Falak-5 and Falak-12 wells, the lower boundary of Bahariya VI corresponds to the well total depth rather than the interpreted top of the Kharita Formation. This increase in thickness is associated with an increase in the net-pay thickness, and the HCPV values that attain their highest values to the northern and southeastern parts revealed at Falak-12, Falak-15.

The core description data is available mainly from the Falak-12 well, whereas the other wells rely primarily on the well-log-based facies interpretation. Core description indicates that the Bahariya Formation consists of four main microfacies: calcareous, silty, glauconitic, and sandy mudstone, representing 43% of the samples; argillaceous sandy siltstone/sandstone, representing 24%; fossiliferous, argillaceous, and glauconitic massive sandstone, representing 29%; and fossiliferous marl and sandy limestone, representing 4%. These lithofacies indicate deposition in a shallow-marine setting, ranging from mixed upper tidal-flat to subtidal-flat environments.

## Results and discussion

The structural setting of the Falak Field was evaluated using the available seismic profiles, which were integrated with formation tops and well-log data to construct a fault–horizon structural framework. Structural-seismic interpretation aims to identify stratigraphic horizons, detect discontinuities caused by faults, and evaluate the fault pattern controlling reservoir geometry. In reservoir characterization, a high-fidelity 3D static model integrates geological observations, geophysical interpretation, and petrophysical measurements to provide a consistent subsurface representation for prospect delineation and field-development planning^1^. The modelling workflow requires careful compilation and quality control of seismic data, well logs, and related reservoir information before constructing the final reservoir framework.

The interpreted seismic horizons and fault geometries were incorporated into the structural model following a standard three-stage workflow. First, the fault model was constructed to define the displacement and geometry of the main deformation elements affecting the target reservoir. Second, a 3D grid framework was generated to establish the master modelling domain. Third, the interpreted horizons were inserted into the grid to subdivide the model into stratigraphic zones and layers, allowing the assessment of structural effects, lithofacies variability, and lateral changes in petrophysical properties^22^.

### Structural modeling

Structural modelling is the first stage in constructing a 3D grid-based reservoir model, as it defines the spatial arrangement of horizons, faults, and reservoir zones. The interpreted horizons and fault surfaces represent the main components of the structural model and provide the framework for subsequent facies and petrophysical property modelling^48,49^. In this study, the Bahariya Formation seismic horizons were interpreted from depth-converted seismic lines and used to build the structural framework of the 3D geological model.

**Fig. 11:**
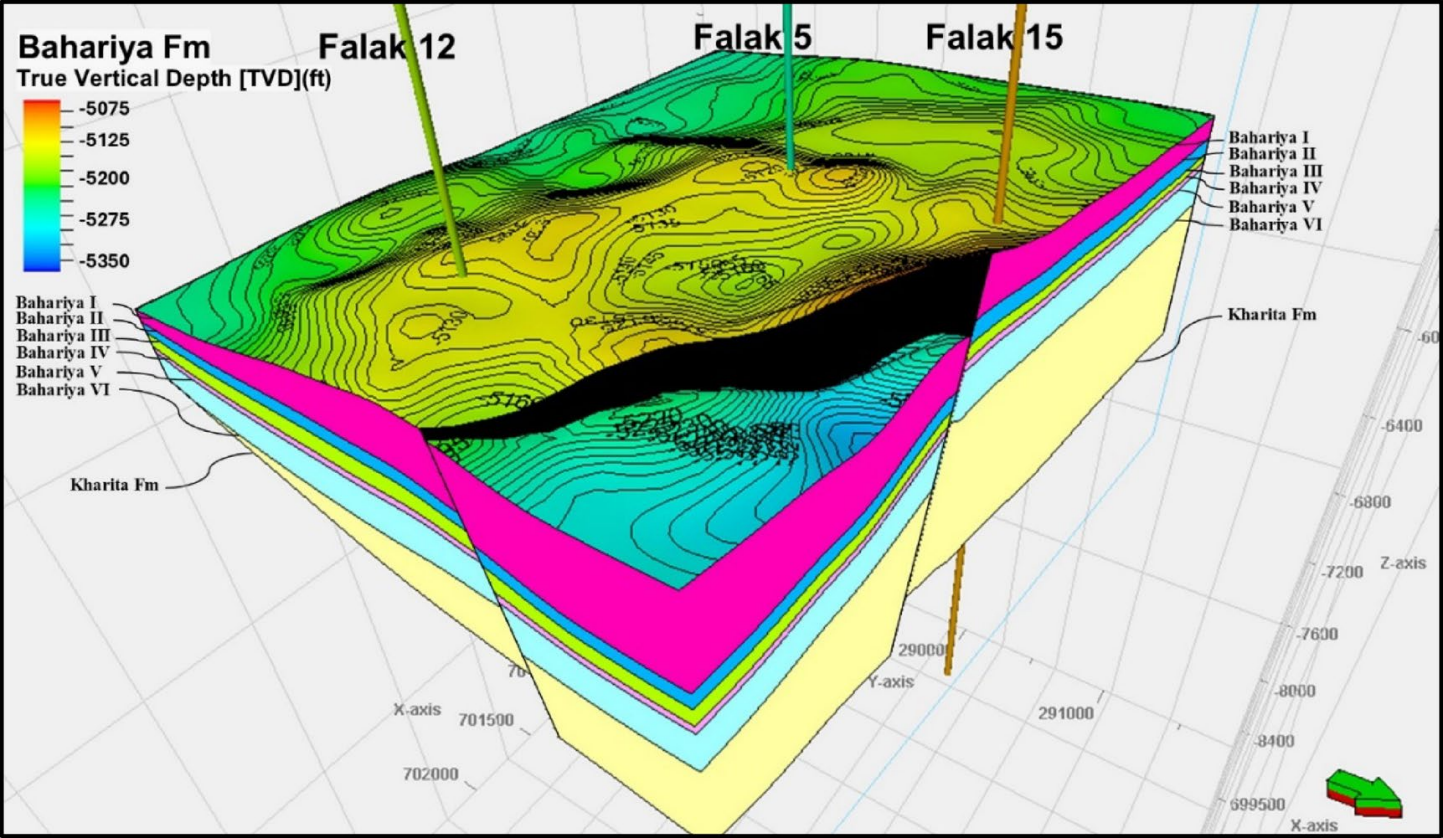
3D Structural model illustrates the vertical depth of top Bahariya Fm in Falak Field.

Horizons and faults were identified and traced to delineate the structural pattern and geometry controlling the Falak Field. The resulting 3D structural grid model incorporates both fault and horizon modelling and illustrates the vertical depth variation of the top Bahariya Formation across the field (Fig. 11). The model shows that the Falak structure is controlled mainly by fault-related deformation, consistent with the interpreted seismic sections and depth-structure maps.

Fault modelling was initiated in Petrel™ by constructing a 3D structural model that ensures geometric consistency between the interpreted seismic horizons and fault surfaces. The faults were modelled primarily from interpreted fault sticks, which were cleaned and trimmed to conform to the reservoir-zone boundaries. Well-log data were correlated with fault-cut depths and integrated into the corresponding faults in the 3D model.

After constructing the fault model, the seismic map was integrated to verify the accuracy of fault placement, orientation, and dip direction within the 3D volume. A 3D grid was then constructed to enclose the fault framework within the defined area of interest.

The structural framework was developed using Petrel’s pillar-gridding technique. This technique generates a 3D lattice by vertically projecting a 2D grid mesh along fault pillars, thereby creating an X, Y, and Z coordinate framework for surface modelling. To maintain cell-shape quality near faults, the zigzag gridding algorithm was used during this stage.

The grid resolution was set to 50 × 50 m to capture the lateral variability observed in the Bahariya reservoir units. Horizon construction was guided by integrating the Bahariya depth grid and formation tops. After construction, the Bahariya horizon was inserted into the 3D grid to define the main stratigraphic boundary for subsequent zonation^50–52^. The stratigraphic sub-zonation was defined according to the depositional characteristics of the Bahariya Formation. The Cenomanian Bahariya Formation is interpreted to have been deposited in a tectonically active setting where palaeo-structural elements influenced sedimentation, resulting in a complex assemblage of fluvio-marine depositional systems.

The static model was constructed using the interpreted formation tops within a conformable stratigraphic framework. Each zone was adjusted after creation to match the selected horizon-generation parameters. The final structural model represents productive reservoir zones that maintain consistent stratigraphic relationships across the closure areas.

Layering was performed to represent small-scale vertical heterogeneity while maintaining a practical number of grid cells for modelling. Each Bahariya zone contains approximately 100 layers. The proportional layering method was applied to generate uniform layer thicknesses from the reservoir top to base. This method provides a relatively simple cell-to-cell configuration suitable for reservoir simulation. With an average layer thickness of approximately 7 ft, the vertical resolution is significantly finer than the 50 × 50 m horizontal grid resolution.

Velocity modelling was then performed using check-shot interval velocity data. The time-depth conversion was carried out using a velocity model based on time-depth ties and check-shot interval velocity information.

###  Property modeling

Property modelling aims to distribute lithofacies and petrophysical properties within the 3D structural grid while preserving the geological and petrophysical heterogeneity observed from well data. In this study, well-log data were integrated with seismic interpretation to quantify the geostatistical distribution of lithofacies and reservoir properties across the stratigraphic units. This stage included reservoir-layer delineation, facies modelling, petrophysical property modelling, and well correlation to ensure consistency with the geological framework.

The interpreted lithofacies and petrophysical properties were assigned to the 3D structural grid to construct property models. Log upscaling was applied to transfer well-log data to the grid scale while preserving the main reservoir-flow characteristics. Reservoir properties, including porosity, shale volume, water saturation, permeability, and related parameters, were modelled using well-log data and geostatistical techniques. Reservoir anisotropy and heterogeneity were considered during the workflow to ensure that the resulting models provide a realistic representation of the subsurface reservoir conditions^44,47,49^.

#### Facies modeling

Facies modelling is used to represent the internal lithological architecture of the reservoir within the 3D grid. While structural modelling defines the external framework of the reservoir, facies modelling describes the spatial distribution of lithological units inside that framework^48,53,54^. Core samples provide the most reliable source of facies information; however, because coring is limited and expensive, well-log interpretation is commonly used to estimate facies in uncored wells.

The facies-modelling workflow began by constructing discrete facies logs. These logs were then upscaled to the grid using the “Most of” averaging method, which is suitable for discrete properties because it assigns the dominant facies to each grid cell. The “Neighbor cell as lines” technique was applied before populating the facies between wells and integrating the results into the 3D grid cells during property simulation. Because each grid cell can contain only one facies value, the interpreted log data were upscaled to assign representative facies values to the model cells. Experimental variogram analysis was then performed to describe the spatial continuity of the facies distribution. After variogram analysis, the Sequential Indicator Simulation algorithm was applied to populate the facies between wells. This method is suitable for modelling discrete categorical variables such as sandstone, siltstone, limestone, and shale.

In the constructed geocellular model, each grid cell was assigned to a dominant lithological value. The Bahariya Formation is predominantly represented by sandstone facies, confirming its role as the main reservoir lithology. This result agrees with regional depositional models that describe the Bahariya Formation as a fluvio-marine to shallow-marine clastic succession composed mainly of sandstones, siltstones, shales, and minor carbonate interbeds^31,55^.

**Fig. 12:**
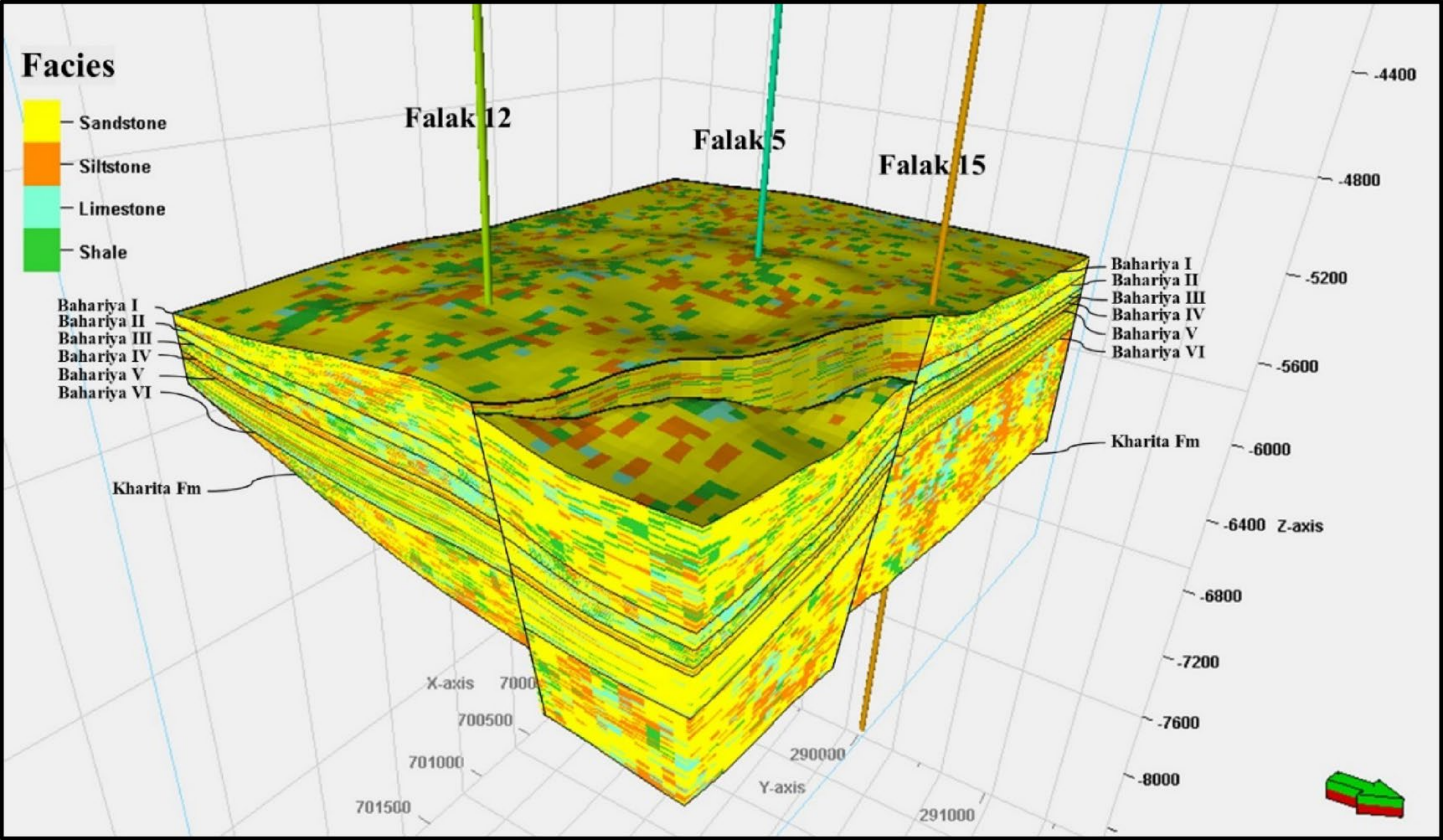
Three-dimensional upscaled Facies model within Falak Field with two intersection in E-W & N-S directions illustrating the lateral and vertical distribution of Facies Types for Bahariya zones.

**Fig. 13:**
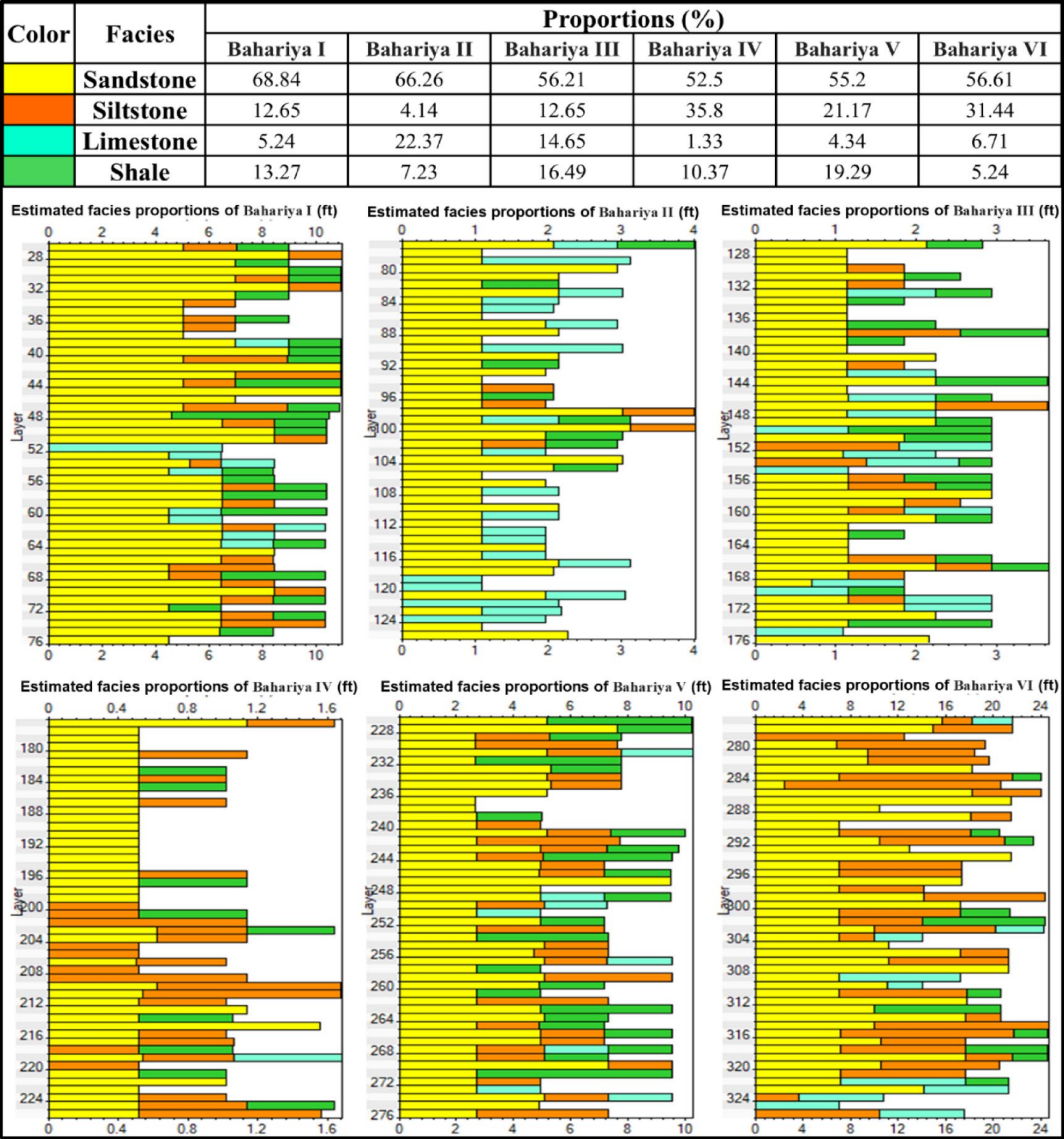
The calculated facies proportions for Bahariya Zones versus the numbered layers within the model.

The facies distribution model (Fig. 12). indicates that sandstone is widely developed across the central and eastern parts of the field, with subordinate siltstone and limestone. The predominance of sandstone reservoir facies suggests that their distribution was controlled mainly by syn-depositional faulting and basin subsidence within the tidal-flat depositional system. Quantitative facies analysis shows that the Bahariya Formation consists of sandstone, siltstone, carbonate, and shale for each zone of Bahariya Reservoir (Fig. 13).

Variogram analysis was used to quantify the three-dimensional spatial variability of facies. The variogram describes the decrease in correlation between data points as separation distance increases. Using the available data, the best-fit variogram models were extracted in the major and minor directions for each reservoir zone. This zone-based variogram approach improves the geological consistency of the facies model and supports the comparison of facies proportions between Bahariya reservoir units.

#### Petrophysical modeling

Petrophysical modelling aims to populate the 3D grid with continuous reservoir properties such as effective porosity, shale volume, water saturation, and permeability. These properties are derived from well-log interpretation and then distributed through the 3D grid using geostatistical methods. In this study, the petrophysical models were constructed after upscaling the interpreted well-log properties to the vertical resolution of the 3D grid. The upscaled properties were constrained by the facies model to preserve the relationship between lithology and reservoir quality^56^.

Before property simulation, experimental variogram analysis was performed to define the spatial continuity and heterogeneity of each petrophysical property. Sequential Gaussian Simulation was then used to model continuous properties such as effective porosity and permeability. Sequential Gaussian Simulation is a stochastic interpolation method based on kriging that is suitable for sparse continuous data. It considers the input data, property distributions, variograms, and trends, while allowing multiple equiprobable realizations to be generated^57^.

Collocated co-kriging was used where appropriate to incorporate correlations between petrophysical properties as secondary constraints during property population^11,48^. Stochastic simulation was then applied within each lithofacies to model the spatial distribution of reservoir properties in a geologically constrained manner. Variogram models were defined following the approach of Deutsch and Journel^58^ to capture spatial continuity and reservoir heterogeneity.

**Fig. 14:**
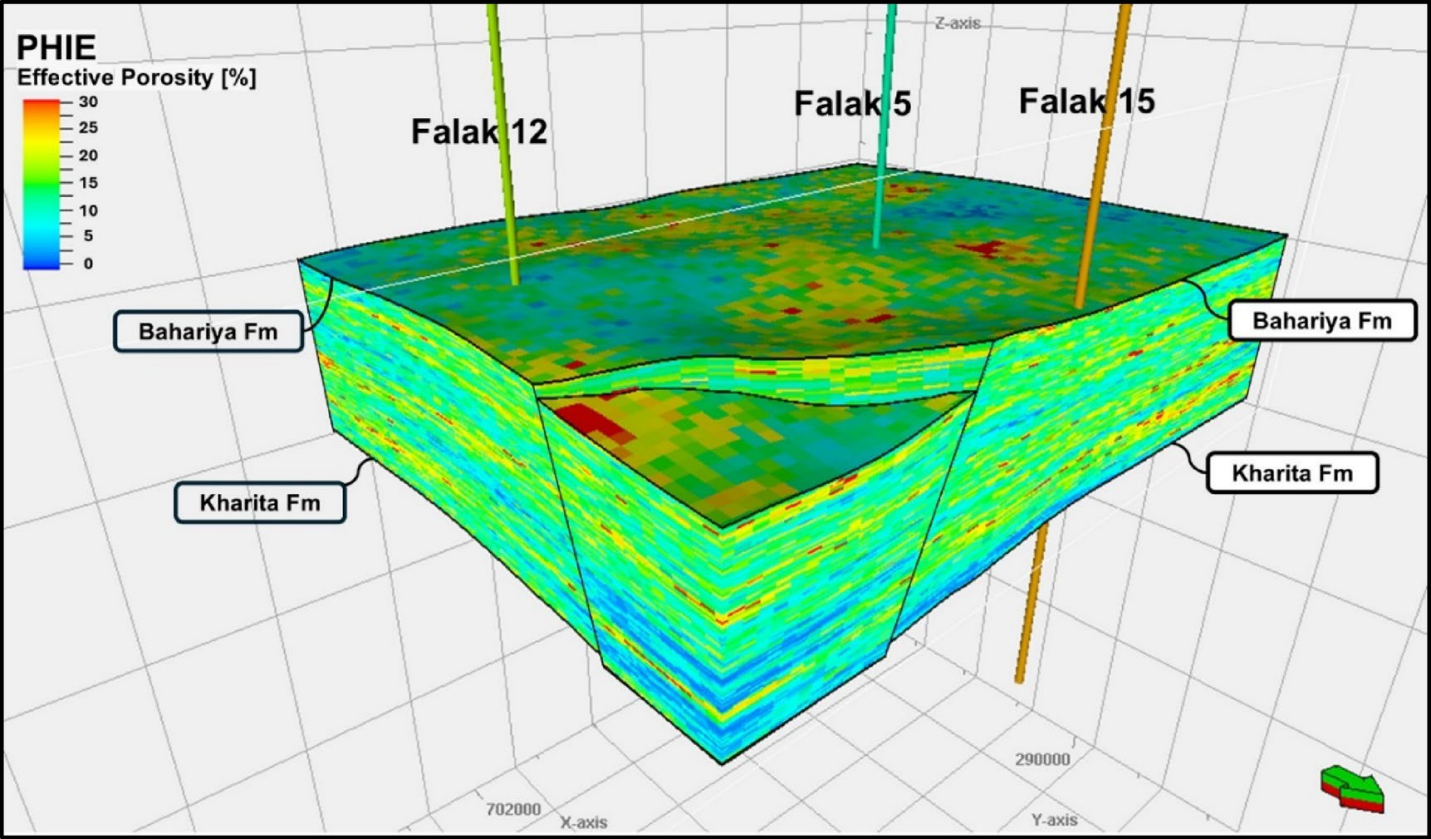
Three-dimensional upscaled effective porosity model within Falak Field with two intersection in E-W & N-S directions.

The effective porosity model shows clear lateral and vertical variation in reservoir quality across the Bahariya Formation (Fig. 14). The central, northwestern, and southeastern parts of the field show better reservoir quality, whereas the southwestern and northeastern parts are characterized by relatively lower effective porosity and higher water saturation, indicating poorer reservoir quality. Vertically, reservoir quality improves toward the lower Bahariya units, reflecting the greater development of fluvial sandstone deposits in the lower part of the formation.

**Fig. 15:**
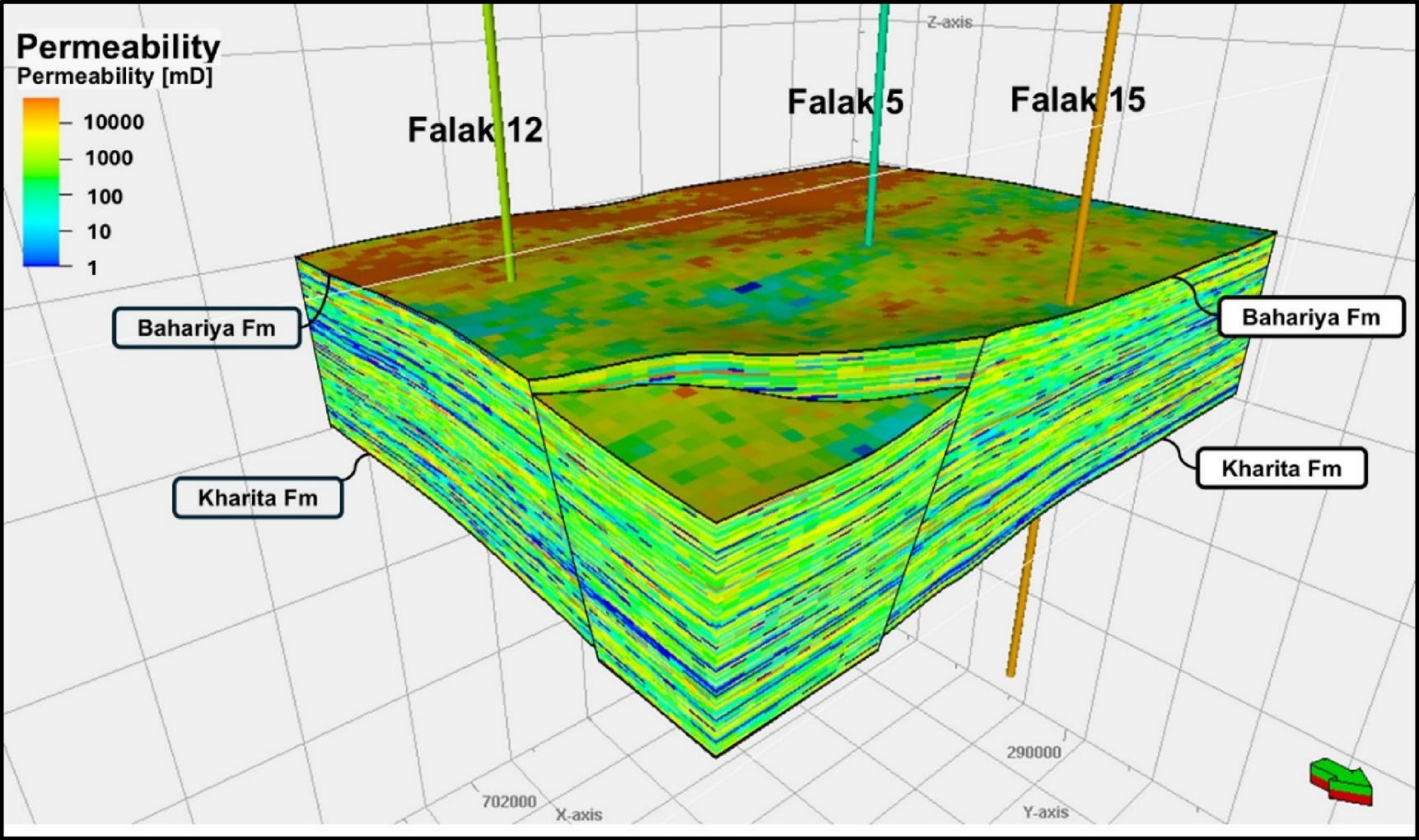
Three-dimensional upscaled permeability model within Falak Field with two intersection in E-W & N-S directions.

The permeability model was generated by integrating the interpreted permeability values with the effective porosity and facies models. The permeability data were first upscaled to the 3D grid, then modelled using Sequential Gaussian Simulation constrained by the facies distribution and variogram parameters. Because permeability is strongly controlled by lithofacies and pore-network connectivity, the model was populated within the facies framework so that sandstone-dominated intervals preserve relatively higher permeability, whereas shale-rich and carbonate-cemented intervals show reduced permeability. The resulting permeability model (Fig. 15) shows higher permeability trends in the central, northwestern, and southeastern parts of the field, consistent with the effective porosity model and the distribution of sandstone facies.

The lateral and vertical variations in petrophysical properties are primarily controlled by facies distribution. The Bahariya Formation is interpreted as a tidal-flat depositional system dissected by south–north-trending channels that prograded seaward into shelfal tidal sands and mudstones^59,60^. This depositional architecture explains the observed heterogeneity in porosity and permeability distribution across the reservoir.

### Reservoir volumetric calculations

Reservoir volumetric assessment represents the final stage of the static reservoir modelling workflow. It provides a quantitative estimation of hydrocarbon resources by integrating the geological model, petrophysical properties, net-to-gross ratio, fluid saturation, and formation volume factor^11,48^. In this study, volumetric calculations were performed using the developed 3D grid-based geocellular model.

The original oil in place was calculated using the following equation

6 $$\:OOIP=\frac{GRV\times\:\phi\:\times\:NTG\times\:{S}_{o}}{{B}_{o}}$$

where $\:{B}_{o}$ is the oil formation volume factor, $\:{S}_{o}$ is oil saturation in fraction, $\:NTG$ is the net-to-gross ratio, $\:OOIP$ is the original oil in place, $\:\phi\:$ is porosity in fraction, and $\:GRV$ is the gross rock volume.

The Bahariya static reservoir model was used to estimate the stock-tank original oil initially in place and to evaluate the overall quality of the Bahariya reservoir units. All input parameters for the volumetric calculation were extracted from the 3D reservoir model, except the oil formation volume factor $\:{B}_{o}$, which was assigned for each reservoir interval and calculated as 1.0642^61–63^. The facies model was used to estimate the net-to-gross ratio by defining the distribution of sandstone, siltstone, shale, and limestone within the Bahariya reservoir units.

Gross rock volume, net rock volume, pore volume, and hydrocarbon pore volume were estimated within the reservoir grid. Pore volume and hydrocarbon pore volume are reservoir-condition volumes, whereas original oil initially in place/stock-tank oil initially in place was calculated by converting hydrocarbon pore volume from reservoir conditions to stock-tank conditions using the oil formation volume factor^64^.

The estimated OOIP for the Bahariya reservoir in the Falak Field is approximately 160 million stock-tank barrels (MMSTB). Of this total, the Bahariya-I unit contributes approximately 46 MMSTB, whereas the Bahariya-III unit accounts for about 114 MMSTB. These volumetric results are supported by hydrocarbon shows and available production data from the wells, confirming the significant oil potential of the Bahariya Formation (Table 3).

**Table 3:** Volumetric calculations for the original oil in place in the Bahariya reservoir units.

Reservoir unit contacts	Bulk volume [MCF]	Net volume [MCF]	Pore volume [MMRB]	HCPV oil [MMRB]	Stock-tank oil initially in place (OOIP)(MMSTB)
Bahariya I	3033	657	151	50	46
Bahariya III	7438	1610	366	122	114
Total	10,471	2267	517	172	160

where MCF refers to million cubic feet, MMRB refers to million reservoir barrels, MMSTB refers to million stock-tank barrels, and OOIP refers to stock-tank oil initially in place.

###  Modeling uncertainty and limitations

To provide a balanced assessment of the study’s findings, this section critically examines the inherent uncertainties, technical challenges, and limitations associated with the 3D static modeling workflow. The analysis focuses on how these factors influence the property distribution models and, consequently, the reliability of the subsequent calculations.

A primary source of uncertainty stems from the inherent limitations of the input dataset. The property distribution is heavily constrained by number of wells, which are non-uniformly distributed across the study area, leading to sparse control in study area^65–67^.

Significant technical challenges were encountered during the property modeling phase. The transformation of well log data (e.g., porosity, permeability, facies) into a 3D grid involved upscaling, which inevitably results in a loss of fine-scale heterogeneity and an averaging effect that can misrepresent the tails of the property distribution (e.g., extremely high-permeability zones)^68–70^.

The methodological approach and the insights derived are not confined to the study area. The integrated workflow combining seismic interpretation, geostatistical property modeling, and uncertainty analysis is directly applicable to analogous geological settings in the region, particularly in Abu Gharadig, Faghur-Siwa, Matruh, Alamein, and Kattaniya Basins where similar structural styles and depositional environments prevail. The demonstrated sensitivity of property distribution to variogram parameters and fault transmissibility assumptions provides a template for de-risking exploration and development activities in structurally complex basins across North Western Desert.

## Conclusions

The heterogeneous clastic Bahariya reservoir in the Falak Field is a structurally controlled by fault compartmentalization, facies variation, and petrophysical quality, where the field is divided by the main NW–SE trending normal fault system and caused the development of two three-way dip closures, representing the most important structural trapping elements in the field.

The reservoir quality of the Bahariya Formation is mainly controlled by the sandstone-dominated intervals. Petrophysical results show that the effective porosity values range from 16.3% to 22.7%. and the oil saturation values range from 44.2% to 51.4%. Facies analysis further shows that sandstone is the dominant lithology (55.48% of the modelled facies), followed by siltstone, shale, and carbonate interbeds.

The 3D static reservoir model shows that the most favourable reservoir-quality zones are located in the central, northwestern, and southeastern parts of the field. Vertically, reservoir quality increases downward in lower Bahariya units, where fluvial sandstone facies are more developed.

Volumetric assessment indicates that the Bahariya reservoir contains approximately 160 MMSTB of OOIP. The Bahariya-III unit represents the main contributor, with approximately 114 MMSTB, whereas the Bahariya-I unit contributes about 46 MMSTB.

Therefore, the northern/northwestern and southeastern structural closures should be considered priority targets for future drilling and field development. The study confirms that integrating seismic interpretation, well-log analysis, facies modelling, petrophysical modelling, and volumetric calculation provides an effective approach for reducing uncertainty and improving reservoir characterization in structurally complex Bahariya reservoirs.

Future research directions are highly recommended, including High-resolution 3D seismic reprocessing and the acquisition of formation micro‐imager (FMI) logs in key wells would reduce structural ambiguity and improve sub‐seismic fracture characterization.

Despite the acknowledged uncertainties, the robust trends identified, particularly the correlation between fault orientation and high-porosity zones, provide a reliable basis for early-stage development planning and well-placement optimization. The presented workflow offers a transferable framework for future static modeling projects in geologically analogous basins.

## Data Availability

The data that support the findings of this study are available from the corresponding author upon reasonable request.

## References

[1] Reda, M. et al. Hydrocarbon reservoir characterization in the challenging structural setting of Southern Gulf of Suez: synergistic approach of well log analyses and 2D seismic data interpretation. 10.20944/preprints202401.0529.v1 (2024).

[2] Dally, N. H. E., Youssef, M. S., Abdel Aal, M. H., Metwalli, F. I. & Nabawy, B. S. Delineating the main structural outlines and the petrophysical properties of the Albian-upper Cretaceous reservoirs using seismic and well log data, Shushan Basin, north Western Desert, Egypt. *J. Pet. Explor. Prod. Technol.* **13**(4), 1009–1030. 10.1007/s13202-022-01603-0 (2023).

[3] Nabawy, B. S. & ElHariri, T. Y. Electric fabric of cretaceous clastic rocks in Abu Gharadig basin, Western Desert, Egypt. *J. Afr. Earth Sc.* **52**(1–2), 55–61. 10.1016/j.jafrearsci.2008.02.003 (2008).

[4] (EGPC), E.G.P.C. *Western Desert, Oil and Gas Fields: A Comprehensive Overview* 431 (Egyptian General Petroleum Corporation, 1992).

[5] Azzam, S. S. S., Elkady, H. H. & Rabea, T. M. M. The impact of seismic interpretation on the hydrocarbon trapping at Falak field, Meleiha, Western Desert, Egypt. *Egypt. J. Pet.* **27**(4), 785–793. 10.1016/j.ejpe.2017.11.006 (2018).

[6] Abdelmaksoud, A. & Radwan, A. A. Integrating 3D seismic interpretation, well log analysis and static modelling for characterizing the Late Miocene reservoir, Ngatoro area, New Zealand. *Geomech. Geophys. Geo-Energy Geo-Resour.* **8**(2), 63. 10.1007/s40948-022-00364-8 (2022).

[7] Nabawy, B. S., Lashin, A. A. & Barakat, M. K. Implementation of lithofacies and microfacies types on reservoir quality and heterogeneity of the Late Cretaceous Upper Bahariya Member in the Shurouk Field, Shoushan Basin, North Western Desert, Egypt. *J. Asian Earth Sci.* **224**, 105014. 10.1016/j.jseaes.2021.105014 (2022).

[8] Abd El-Hay, M. et al. Integrated seismic-stratigraphic, sedimentological and petrophysical approaches for characterizing the reservoir potential of early Cretaceous Alam El Bueib formation in the Meleiha concession of North Western Desert, Egypt. *Mar. Pet. Geol.* **160**, 106659. 10.1016/j.marpetgeo.2023.106659 (2024).

[9] Bayoumi, T. The influence of interaction of depositional environment and synsedimentary tectonics on the development of some Late Cretaceous source rocks, Abu Gharadig Basin, Western Desert, Egypt. In *Proceedings of the EGPC 13th Petroleum Exploration & Production Conference, Cairo*, Vol. 2, 475–496 (1996).

[10] Richardson, S. M., Vivian, N., Cook, R. J., Wilkes, M. & Hussein, H. Application of fault seal analysis techniques in the Western Desert, Egypt. *Geol. Soc. Lond. Spec. Publ.* **132**(1), 297–315. 10.1144/GSL.SP.1998.132.01.17 (1998).

[11] Abdel-Fattah, M. I., Metwalli, F. I. & El Sayed, I. M. Static reservoir modeling of the Bahariya reservoirs for the oilfields development in South Umbarka area, Western Desert, Egypt. *J. Afr. Earth Sci.* **138**, 1–13. 10.1016/j.jafrearsci.2017.11.002 (2018).

[12] Abdelwahhab, M. A. & Raef, A. Integrated reservoir and basin modeling in understanding the petroleum system and evaluating prospects: The Cenomanian reservoir, Bahariya Formation, at Falak Field, Shushan Basin, Western Desert, Egypt. *J. Pet. Sci. Eng.* **189**, 107023 (2020).

[13] Hassan, T. et al. An advanced workflow to compress the uncertainties of stochastic distribution of Bahariya reservoir properties using 3D static modeling: An example from Heba Oil Fields, Western Desert, Egypt. *Pet. Res.*10.1016/j.ptlrs.2022.09.001 (2023).

[14] Meshref, W. M. *Tectonic framework of Egypt* (Balkema, 1990).

[15] Said, R. Tectonic framework of Egypt. In *The Geology of Egypt* 28–44 (Elsevier, 1962).

[16] Schandelmeier, H., Klitzsch, E., Hendriks, F. & Wycisk, P. Structural development of north-east Africa since Precambrian times. http://pascal-francis.inist.fr/vibad/index.php?action=getRecordDetail&idt=7476924 (1987).

[17] Guiraud, R. et al. Phanerozoic geological evolution of Northern and Central Africa: an overview. *J. Afr. Earth Sci.* **43**(1–3), 83–143. 10.1016/j.jafrearsci.2005.07.017 (2005).

[18] Bayoumi, A. & Lotfy, H. Modes of structural evolution of Abu Gharadig Basin, Western Desert of Egypt as deduced from seismic data. *J. Afr. Earth Sci.* **9**(2), 273–287. 10.1016/0899-5362(89)90070-5 (1989).

[19] Keeley, M. The Jurassic System in northern Egypt: II. Depositional and tectonic regimes. *J. Petroleum Geol.* **14**(1), 49–64. 10.1111/J.1747-5457.1991.TB00298.X (1991). *Martin L.*Wallis, R.

[20] El Shazly, E. M. The geology of the Egyptian region. In *the ocean basins and margins: Volume 4A The Eastern Mediterranean* 379–444 (Springer, 1977). 10.1007/978-1-4684-3036-3_10.

[21] Dolson, J. C. et al. The petroleum potential of Egypt. 10.1306/M74775C23 (2001).

[22] Barakat, M. K., El-Gendy, N. H. & El-Bastawesy, M. A. Structural modeling of the Alam EL-Bueib Formation in the Jade oil field, Western Desert, Egypt. *J. Afr. Earth Sci.* **156**, 168–177. 10.1016/j.jafrearsci.2019.05.003 (2019).

[23] Darwish, M. et al. Sedimentology, environmental conditions and hydrocarbon habitat of the Bahariya Formation, central Abu Gharadig basin, Western Desert, Egypt. In *EGPC 12th Petroleum Exploration and Production Conference* (1994).

[24] Nabawy, B. S., Abd El Aziz, E. A., Ramadan, M. & Shehata, A. A. Implication of the micro- and lithofacies types on the quality of a gas-bearing deltaic reservoir in the Nile Delta, Egypt. *Sci. Rep.* **13**(1), 8873 (2023). 37264046 10.1038/s41598-023-35660-0 PMC10235052

[25] Schlumberger. *Well Evaluation Conference, Egypt.* 87 (Technical Editing Services, Ltd., 1995).

[26] Schlumberger, P. Geology of Egypt. In *Chapter I, Well Evaluation Conference* (1984).

[27] Lei, Z. et al. A semi-analytical model of a hydraulically fractured horizontal well with pre-Darcy flow and stimulated reservoir volume in a radial composite shale reservoir. *SPE J.* **30**(02), 743–761. 10.2118/223942-PA (2025).

[28] Chirkin, I. et al. Combining seismic waves of different classes to enhance the efficiency of seismic exploration. In *SEG International Exposition and Annual Meeting* (SEG, 2016).

[29] Elmahdy, M. et al. Integrated geophysical, petrophysical and petrographical characterization of the carbonate and clastic reservoirs of the Waihapa Field, Taranaki Basin, New Zealand. *Mar. Pet. Geol.* **151**, 106173. 10.1016/j.marpetgeo.2023.106173 (2023).

[30] Haque, A. E. et al. Integrated wireline log and seismic attribute analysis for the reservoir evaluation: A case study of the Mount Messenger Formation in Kaimiro Field, Taranaki Basin, New Zealand. *J. Nat. Gas Sci. Eng.* **99**, 104452. 10.1016/j.jngse.2022.104452 (2022).

[31] Nabawy, B. S. et al. Seismic reservoir characterization of the syn-rift lower Miocene Rudeis Formation in the July oilfield, Gulf of Suez basin, Egypt: implication for reservoir quality assessment. *Geoenergy Sci. Eng.* **26**, 211797. 10.1016/j.geoen.2023.211797 (2023).

[32] White, R. & Simm, R. Tutorial: Good practice in well ties. *First Break*10.3997/1365-2397.21.10.25640 (2003).

[33] Abdeen, M. M. et al. Subsurface structural setting and hydrocarbon potentiality of the Komombo and Nuqra Basins, South Egypt: A seismic and petrophysical integrated study. *Nat. Resour. Res.* **30**(5), 3575–3603. 10.1007/s11053-021-09898-2 (2021).

[34] Baouche, R. & Nabawy, B. S. Permeability prediction in argillaceous sandstone reservoirs using fuzzy logic analysis: A case study of Triassic sequences, Southern Hassi R’Mel Gas Field, Algeria. *J. Afr. Earth Sci.* **173**, 104049. 10.1016/j.jafrearsci.2020.104049 (2021).

[35] Cherana, A., Aliouane, L., Doghmane, M. Z., Ouadfeul, S.-A. & Nabawy, B. S. Lithofacies discrimination of the Ordovician unconventional gas-bearing tight sandstone reservoirs using a subtractive fuzzy clustering algorithm applied on the well log data: Illizi Basin, the Algerian Sahara. *J. Afr. Earth Sci.* **196**, 104732 (2022).

[36] Radwan, A. A. et al. Facies analysis-constrained geophysical 3D-static reservoir modeling of Cenomanian units in the Aghar Oilfield (Western Desert, Egypt): insights into paleoenvironment and petroleum geology of fluviomarine systems. *Mar. Pet. Geol.* **136**, 105436. 10.1016/j.marpetgeo.2021.105436 (2022).

[37] Feng, P. et al. Spatio-temporal evolution of multiple water bodies and a water conservation mining strategy in the Xinjiang coalfield: A case study of the Yushuquan and Yongxin mines. *Mine Water Environ.* **44**(3), 694–708. 10.1007/s10230-025-01066-9 (2025).

[38] Larionov, V. J. N. Moscow, Borehole radiometry. Vol. 127, 813 (1969).

[39] Asquith, G. B. & Gibson, C. R. *Basic well log analysis for geologists* Vol. Vol. 3 (American Association of Petroleum Geologists, 1982). 10.1306/Mth3425.

[40] Ren, Q. et al. An innovative approach to discrete facture network modeling driven by geomechanics and multiple factors. *Geoenergy Sci. Eng.* **257**, 214200. 10.1016/j.geoen.2025.214200 (2025).

[41] Xu, Z. et al. BrIMs-based 3D semantic segmentation of bridge components leveraging multisensor fusion. *J. Comput. Civ. Eng.* **40**(3), 4026012. 10.1061/JCCEE5.CPENG-7258 (2026).

[42] Zhang, Q., Zhang, K., Chao, L., Chen, X. & Wu, N. A unified runoff generation scheme for applicability across different hydrometeorological zones. *Environ. Model. Softw.* **180**, 106138. 10.1016/j.envsoft.2024.106138 (2024).

[43] Xue, S. et al. Visualization characterization of void structure evolution in broken limestone and its influence on permeability. *Fuel* **405**, 136412. 10.1016/j.fuel.2025.136412 (2026).

[44] Doghmane, M. Z. et al. A new approach for estimating water saturation in low-resistivity hydrocarbon-bearing reservoirs using artificial neural network (ANN). *Neural Comput. Appl.* **37**(6), 4409–4437 (2025).

[45] Shehata, A. A. et al. Sequence stratigraphic evolution of the syn-rift early Cretaceous sediments, West Beni Suef Basin, the Western Desert of Egypt with remarks on its hydrocarbon accumulations. *Arab. J. Geosci.* **11**(12), 331. 10.1007/s12517-018-3688-y (2018).

[46] Shehata, A. A. et al. Senonian platform-to-slope evolution in the tectonically-influenced Syrian Arc sedimentary belt: Beni Suef Basin, Egypt. *J. Afr. Earth Sci.* **170**, 103934. 10.1016/j.jafrearsci.2020.103934 (2020).

[47] El-Din, E. S. et al. Assessment of petrophysical parameters of clastics using well logs: The Upper Miocene in El-Wastani gas field, onshore Nile Delta, Egypt. *Pet. Explor. Dev.* **40**(4), 488–494. 10.1016/S1876-3804(13)60062-2 (2013).

[48] Cannon, S. *Reservoir Modelling: A Practical Guide* (Wiley Publishing, 2018).

[49] Cosentino, L. *Integrated reservoir studies* (Editions Technip, 2001).

[50] Cao, C. et al. Transport mechanisms of CO2/N2-CH4 flow: A digital rock study based on in-situ reaction of CO2-brine-carbonate systems. *Energy* **344**, 139772. 10.1016/j.energy.2025.139772 (2026).

[51] Song, W. et al. DynAvatar: Dynamic 3D head avatar deformation with expression guided gaussian splatting. *IEEE Trans. Vis. Comput. Graph.* 1–13. 10.1109/TVCG.2025.3640423 (2025). 10.1109/TVCG.2025.3640423 41343319

[52] Zhao, Y. et al. Characterizing uncertainty in process-based hydraulic modeling, exemplified in a semiarid Inner Mongolia steppe. *Geoderma* **440**, 116713. 10.1016/j.geoderma.2023.116713 (2023).

[53] Liu-Zeng, J. et al. Fault orientation trumps fault maturity in controlling coseismic rupture characteristics of the 2021 Maduo earthquake. *AGU Adv.* **5**(2), e2023AV001134. 10.1029/2023AV001134 (2024).

[54] Shen, X. et al. Late Miocene extension of the Dali fault system in Southeast Tibet: Coupled tectonic inversion and landscape evolution. *GSA Bull.*10.1130/B38058.1 (2025).

[55] Li, G. et al. Thawing permafrost under Qinghai-Xizang Highway and its impacts on road performance based on multi-source observed data. *Cold Reg. Sci. Technol.* **244**, 104828. 10.1016/j.coldregions.2026.104828 (2026).

[56] Abdel-Fattah, M., Dominik, W., Shendi, E., Gadallah, M. & Rashed, M. 3D integrated reservoir modelling for upper safa gas development in Obaiyed field, Western Desert, Egypt. In *72nd EAGE Conference and Exhibition Incorporating SPE EUROPEC 2010*, cp-161. 10.3997/2214-4609.201401358 (European Association of Geoscientists & Engineers, 2010).

[57] Liu, Z. et al. Impact of enhanced high-latitude moisture transport on precipitation in Central Arid Region of Asia: Evidence from stable isotopes in precipitation. *Atmos. Res.* **339**, 109040. 10.1016/j.atmosres.2026.109040 (2026).

[58] Yang, J. et al. Global soil water stable isotope dataset. *Sci. Data*. 10.1038/s41597-026-07262-8 (2026). 42000782 10.1038/s41597-026-07262-8 PMC13276124

[59] Catuneanu, O., Khalifa, M. A. & Wanas, H. J. S. G. Sequence stratigraphy of the lower Cenomanian Bahariya formation, Bahariya oasis, Western desert, Egypt. *Sediment. Geol.* **190**(1–4), 121–137. 10.1016/j.sedgeo.2006.05.010 (2006).

[60] Metwalli, F., Bakr, A. & Vision, T. Seismostratigraphic analysis of the Alam El Bueib reservoir sand, south umbarka area, Western Desert, Egypt. *ISESCO Sci. Technol. Vis.* **3**, 64–87 (2007).

[61] Rejas Rasheed, P. & Kulkarni, A. Reserve estimation using volumetric method. *Int. Res. J. Eng. Technol. (IRJET)*. **3**, 1225–1229 (2016).

[62] Song, W. et al. Expressive 3D facial animation generation based on local-to-global latent diffusion. *IEEE Trans. Vis. Comput. Graph.* **30**(11), 7397–7407. 10.1109/TVCG.2024.3456213 (2024). 39255115 10.1109/TVCG.2024.3456213

[63] Sun, M. et al. Swin-UNETR: A transformer-based model for 3D pore network segmentation in low-permeability sedimentary rocks. *Int. J. Coal Geol.* **315**, 104951. 10.1016/j.coal.2026.104951 (2026).

[64] Dai, D. et al. Probabilistic prediction and uncertainty for underwater 3-D reconstruction. *IEEE Trans. Instrum. Meas.* **75**, 1–17. 10.1109/TIM.2026.3666010 (2026).

[65] Su, Q. et al. LeF-MTP: Prioritizing GNN test cases by fusing model uncertainty and feature-space confusability. *Comput. Intell.* **42**(1), e70199. 10.1111/coin.70199 (2026).

[66] Xu, Z. et al. Oil-source correlation and controlling effects of oil shale on tight oil accumulation in the Triassic Chang 7 member, Longdong area, Ordos basin. *Pet. Geosci.* petgeo2025-petgeo2076. 10.1144/petgeo2025-076 (2026).

[67] Xu, S. et al. Sedimentary evolution and shale oil potential of Shahejie formation in Zhanhua sag, Bohai Bay basin, China. *AAPG Bull.* **110**(2), 115–141. 10.1306/11122524007 (2026).

[68] Liu, G. et al. Three-dimensional (3D) laser scanning–based identification of rock mass discontinuities for rockfall modeling using 3D discontinuous deformation analysis. *Int. J. Rock Mech. Min. Sci.* **202**, 106484. 10.1016/j.ijrmms.2026.106484 (2026).

[69] Pei, Z. et al. Multiscale anisotropic mechanical properties of oil shale: New insights from nanoindentation profiling. *Pet. Sci.* **23**(1), 33–51. 10.1016/j.petsci.2025.09.011 (2026).

[70] Zhao, H. et al. Airport runway roughness evaluation using TCP-InSAR technology. *Int. J. Pavement Eng.*10.1080/10298436.2025.2496335 (2025).

